# Emotional Distress During COVID-19 by Mental Health Conditions and Economic Vulnerability: Retrospective Analysis of Survey-Linked Twitter Data With a Semisupervised Machine Learning Algorithm

**DOI:** 10.2196/44965

**Published:** 2023-03-16

**Authors:** Michiko Ueda, Kohei Watanabe, Hajime Sueki

**Affiliations:** 1 Department of Public Administration and International Affairs The Maxwell School of Citizenship and Public Affairs Syracuse University Syracuse, NY United States; 2 Center for Policy Research The Maxwell School of Citizenship and Public Affairs Syracuse University Syracuse, NY United States; 3 Waseda Institute for Advanced Study Waseda University Tokyo Japan; 4 Faculty of Human Sciences Wako University Tokyo Japan

**Keywords:** mental health, COVID-19, Twitter, social media, depression, suicidal ideation, loneliness, public health crisis, psychological well-being, infodemiology, machine learning framework, digital surveillance, emotional distress, online survey

## Abstract

**Background:**

Monitoring the psychological conditions of social media users during rapidly developing public health crises, such as the COVID-19 pandemic, using their posts on social media has rapidly gained popularity as a relatively easy and cost-effective method. However, the characteristics of individuals who created these posts are largely unknown, making it difficult to identify groups of individuals most affected by such crises. In addition, large annotated data sets for mental health conditions are not easily available, and thus, supervised machine learning algorithms can be infeasible or too costly.

**Objective:**

This study proposes a machine learning framework for the real-time surveillance of mental health conditions that does not require extensive training data. Using survey-linked tweets, we tracked the level of emotional distress during the COVID-19 pandemic by the attributes and psychological conditions of social media users in Japan.

**Methods:**

We conducted online surveys of adults residing in Japan in May 2022 and collected their basic demographic information, socioeconomic status, and mental health conditions, along with their Twitter handles (N=2432). We computed emotional distress scores for all the tweets posted by the study participants between January 1, 2019, and May 30, 2022 (N=2,493,682) using a semisupervised algorithm called latent semantic scaling (LSS), with higher values indicating higher levels of emotional distress. After excluding users by age and other criteria, we examined 495,021 (19.85%) tweets generated by 560 (23.03%) individuals (age 18-49 years) in 2019 and 2020. We estimated fixed-effect regression models to examine their emotional distress levels in 2020 relative to the corresponding weeks in 2019 by the mental health conditions and characteristics of social media users.

**Results:**

The estimated level of emotional distress of our study participants increased in the week when school closure started (March 2020), and it peaked at the beginning of the state of emergency (estimated coefficient=0.219, 95% CI 0.162-0.276) in early April 2020. Their level of emotional distress was unrelated to the number of COVID-19 cases. We found that the government-induced restrictions disproportionately affected the psychological conditions of vulnerable individuals, including those with low income, precarious employment, depressive symptoms, and suicidal ideation.

**Conclusions:**

This study establishes a framework to implement near-real-time monitoring of the emotional distress level of social media users, highlighting a great potential to continuously monitor their well-being using survey-linked social media posts as a complement to administrative and large-scale survey data. Given its flexibility and adaptability, the proposed framework is easily extendable for other purposes, such as detecting suicidality among social media users, and can be used on streaming data for continuous measurement of the conditions and sentiment of any group of interest.

## Introduction

### Background

As a global health crisis, the COVID-19 pandemic presented unprecedented challenges to policy makers worldwide. Not only did the disease pose a major threat to the physical health of the public, it also constituted a source of significant psychological distress caused by the ramifications of the disease itself and government actions to contain the spread of the disease, which included city/community lockdowns, school closure, and movement restrictions.

During rapidly developing public health crises, such as the COVID-19 pandemic, continuous monitoring of the psychological well-being of the population is crucial. However, the real-time surveillance of the psychological health of the population remains challenging, mainly due to the lack of suitable data and techniques that can be used for monitoring purposes. Traditional public health surveillance methods, including those that use hospital and death records, typically suffer from a time lag in reporting. They also lack detailed information about the patients or the deceased, such as their socioeconomic status. Well-designed, large-scale survey data can reveal the psychological conditions of the population, but they are not suitable for continuous monitoring, because conducting frequent surveys for a prolonged period is not feasible.

A more recent approach uses digital traces, such as social media posts, to understand the sentiment and conditions of the general population or social media users [1]. This approach has gained popularity during the COVID-19 pandemic as a relatively easy and cost-effective option, resulting in many studies that have analyzed the contents of Twitter data to understand the sentiment and psychological well-being of social media users [2-8]. Although they provide a powerful framework, the identity and attributes of individuals who created these posts are largely unknown. Accordingly, it is difficult to gain insights into which segment of the social media users is significantly affected by the crisis and its associated disruptions; for example, it remains unknown from previous studies that have analyzed tweets whether individuals in financial distress were affected more severely by the pandemic compared to those in financial stability. Methods to impute the attributes of social media users have been proposed, but they are limited to basic demographic information, such as age group and gender [9].

### Aims

The aims of this study were twofold. First, we proposed a machine learning framework for the real-time surveillance of mental health conditions that does not require extensive training data. Second, using survey-linked tweets, we tracked the level of emotional distress of social media users in Japan during the COVID-19 pandemic by their characteristics and psychological conditions. We hypothesized that the emotional distress level of social media users increased when (1) the number of infections increased; (2) the amount of COVID-19–related news increased, as it might have added to their anxiety and fear; (3) the government introduced major social restrictions; and (4) when the media reported the deaths by suicide of celebrities. The last hypothesis was derived because findings from previous studies strongly suggest that the number of suicides tends to increase after media reports on prominent suicides [10,11] and also that Twitter users react emotionally to these reports [12,13]. There were 2 instances of prominent celebrity suicides reported in Japan in 2020. The first was in mid-July by a 30-year-old actor, and the second was in late September by a 40-year-old actress.

Twitter was an appropriate data source for this study. Twitter is one of the most popular social media platforms in Japan, and the number of active users in Japan is estimated to be around 60 million, amounting to almost half of the country’s population, and is ranked second in the world [14].

### COVID-19 in Japan

Our study focused on the first year of the COVID-19 pandemic (2020) and 2019 as a baseline period. The first case of COVID-19 in Japan was confirmed on January 15, 2020, which was followed by the news of a cruise ship anchored at the port of Yokohama with a cluster of COVID-19 cases on board in early February. School closure started on March 2, 2020, and lasted for about 3 months. The first declaration of a state of emergency was made on April 7, 2020. No lockdown was in place during the state of emergency. The state of emergency was lifted on May 25, 2020, and there was no subsequent emergency declaration until January 2021. Life briefly returned to normalcy by the early summer in 2020. Movement restrictions across prefecture boundaries were lifted on June 19, and the government started subsidy programs to encourage domestic travel on July 22, which lasted until December 28, 2020. The country’s average stringency index in March-December 2020 was 36.66 out of 100, which is lower than the average index during the same period in other G7 countries, which was 66.50 [15]. The total number of COVID-19 cases and deaths in Japan as of December 31, 2020, was 1901.94 and 28.17 per 1 million persons, respectively, whereas the corresponding numbers in the rest of the G7 countries were 34588.96 and 909.79 per 1 million persons, respectively [15].

## Methods

### Survey

We conducted anonymous online surveys of adults residing in Japan on May 8 and 24-28, 2022, as part of a larger project. We asked a commercial survey company, the Survey Research Center, to recruit samples from its pool of registered participants, which is the largest in Japan with more than 2 million individuals.

The survey was conducted in 2 steps. First, the survey company sent out a short questionnaire on Twitter usage to its pool of participants residing in Japan and aged 18 years or older. Those who answered that they regularly upload posts on their public Twitter account and those who provided explicit consent to our request to download their past and future tweets for research purposes were then asked to provide their Twitter handles (account names), which concluded the first part of the survey. We then verified the existence of each user’s account and the frequency of its usage using Twitter’s application programming interface (API). Only those with a legitimate account were invited to participate in the second part of the survey. In the second part, we asked for the participants’ basic demographic information, socioeconomic status, and mental health conditions and loneliness (more details later). The survey company prescreened responses with invalid and incomplete responses, and thus, there were no missing data in the survey. The initial size of our sample was 2432.

We excluded 729 participants who failed 2 attention check questions included in the survey; failure indicates a lack of commitment to the tasks. Other exclusion criteria based on the participants’ Twitter usage are explained in the Twitter data collection subsection later.

### Ethical Considerations

The participants provided explicit consent in an online form regarding their participation in the survey and the retrieval of past and future tweets (from January 1, 2019, to June 30, 2022). The survey was anonymous, and the participants were allowed to exit from the survey anytime by closing the browser. Upon completion, they received a small amount of monetary compensation from the survey company. The study was approved by the Ethics Committee of Wako University (approval number: 2022-004).

### Measures

The main survey questions asked the participants their age group, sex, annual household income (in JPY 2 million, US $14,850.40, increments), employment status, and type of employment, if employed. Other demographic information, such as marital status and the area of residency, was included in the questionnaire but not used in this study. The participants were grouped into 3 age groups (ages 18-29, 30-39, and 40-49 years). In this study, we restricted the participants’ age to between 18 and 49 years because older generations are less likely to actively use Twitter [16]. In addition, old individuals who actively use Twitter are less likely to be representative of the elderly population. We excluded 737 (29.98%) participants based on the age criterion from the analysis.

For the income group, we divided the sample into 2 groups: those whose annual household income is below JPY 6 million (US $ 44,551.20 as of November 2022) or equivalent and those who annual household income is ≥JPY 6 million. As of 2020, Japan’s median and average household incomes were JPY 4.4 million (US $ 32,670.88) and JPY 5.6 million (US $41,581.12), respectively [17].

The employment status and type of employment were combined into 1 variable, and the participants were categorized as “permanent employee,” “part-time/temporary worker or unemployed,” or “not in the labor force.” The second category includes those employed as short-term contract workers, dispatch workers, or part-time/hourly workers and those actively looking for a job. Participants who did not fall into any of these 3 categories, such as those who selected “other” in the employment status, were excluded from the analysis when we stratified the data by employment status and type.

We measured the participants’ level of depressive symptoms with the 9-item Patient Health Questionnaire (PHQ-9) [18]. The total score for the PHQ-9 ranges from 0 to 27, and we categorized our participants into 2 groups, “no depressive symptoms” and “with depressing symptoms,” using a score of 10 and higher as a cut-off for the latter, as recommended [19]. Cronbach α of the sample was .91.

We also measured the level of suicidal ideation with the last item (item 9) of the PHQ-9. The item asks how often the individual is bothered by thoughts that they would be better off dead or hurting themselves in some way over the past 2 weeks. The response to item 9 is shown to be associated with actual suicide risk [20,21]. We grouped the participants into 2 groups: “no suicidal ideation” (those who answered “not at all”) and “with suicidal ideation” (those who answered “several days,” “more than half the days,” or “nearly every day”).

The level of loneliness was measured with the 3-item loneliness scale [22]. The total score ranges from 3 to 9, and the participants were categorized into 2 groups using 6 or more as a cut-off for the category “lonely,” following previous studies [23,24].

It should be noted that the participants’ characteristics and conditions were measured in May 2022, and thus, they may have been different in 2019 and 2020. In particular, it is possible that the mental health conditions of some participants had been worse during the peak period of the pandemic, which might have resulted in some of these individuals being miscategorized in the “no depressive symptoms” category, for instance, when in fact they had such psychological symptoms back in 2020 (see also the Discussion section later).

### Data

#### News Reports

We also collected data on the number of COVID-19–related news reports in 2020 and the number of reported COVID-19 cases to examine how the volume of media reporting on COVID-19 and the severity of COVID-19 infection rates are related to the level of emotional distress. The data on news coverage were taken from a website created by the Nippon Hoso Kyokai (NHK; national public media), which archives all of its past news related to COVID-19 [25], and we computed weekly counts of news reporting. We did not distinguish domestic and international events. The first report on COVID-19 was made on January 5, 2020, regarding a case of pneumonia with an unknown cause reported in Wuhan, China. The weekly total of new confirmed cases was calculated from the Our World in Data website [15].

#### Twitter Posts

We collected the participants’ full historical public posts on Twitter since January 2019 using a Twitter API and an academic developer account, which allowed us to download public posts of specific users without restrictions.

We retrospectively collected all the tweets posted by all our survey participants between January 1, 2019, and May 30, 2022 (N=2,493,682). We then computed a distress score for each tweet using a semisupervised algorithm called latent semantic scaling (LSS) [26]. It was an ideal content analysis method for the purpose of this study because it is infeasible to create a keyword dictionary to detect all signs of mental distress or to select a large number of such tweets to train supervised machine learning algorithms (eg, neural networks).

LSS recognizes the semantics of all other words, including emojis in the tweets, based on a few “seed words” that characterize the state of emotional distress. The algorithm achieves this by computing the semantic similarity between the seed words and all other words in the corpus. In selecting seed words for emotional distress, we first created a list of candidate words related to the negative mental status using the *Japanese Emotional Expression Dictionary* [27] and then fitted an LSS model with each of them to manually inspect their semantics. We discarded candidate words that had high similarity with words that were not necessarily related to emotional distress before finalizing the seed words (Table 1). In computing the semantic similarity between words, we included all the tweets we collected between 2019 and 2022 to inform the algorithm as much as possible.

**Table 1:** Seed words for emotional distress.

Status	Japanese	English (translation)
Worse	悩み, 心配, 嘆く, 泣く, 悔しい, 困る, しんどい, 苦痛	worry, lament, cry, regret, trouble, annoyance, distress, painful
Better	楽しみ, 絶好調, 喜ぶ, 笑う, 嬉しい, 幸せ, のんびり, 元気	fun, great, delighted, laugh, happy, relaxed, energetic

#### Algorithm of LSS

LSS performs singular vector decomposition (SVD) of a document-feature matrix to estimate the semantic proximity of words. Mathematically, the method is similar to other dimension reduction techniques, such as factor analysis. SVD is first applied to a document-term matrix *D*,



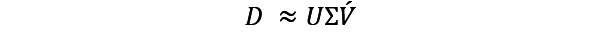



where 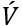 contains word vectors *v_1_,…v_f_* for all the words. Next, the average similarity *g_1_,…g_f_* between word vectors for seed words in S is computed to create polarity scores for all the words, *p_s_*:



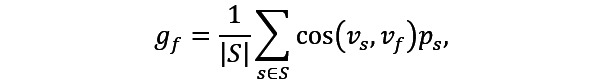



where *cos(v_x_, v_y_)* is the cosine similarity between the word vector of words *x* and *y*. We set the document dimension to 200 to capture more basic semantics of words in SVD. We computed the polarity score of a tweet *k*, which comprises features *F*, by taking the sum of the polarity scores *g_f_* weighted by the frequency of words *h_f_*:



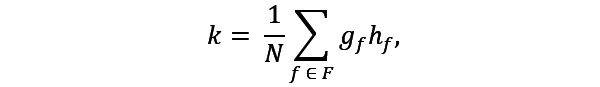



where *N* is the total number of words in the tweet. The polarity scores are recentered to the overall mean of the corpus and rescaled by the SD of the scores. This rescaling makes the scores easier to interpret as they distribute around 0 with SD=1.

Finally, we identified posts by inactive users, posts not in Japanese, those that only contained links to popular news sites, and retweets. After excluding users whose posts consisted of only such posts (N=406, 16.69%), we were left with a corpus of 495,021 (19.85%) tweets generated by 560 (23.03%) individuals in 2019 and 2020. Of the 495,021 tweets in the final data set, 488,284 (98.64%) were assigned an LSS score and used in the subsequent analysis.

#### Validation of LSS by Human Coders

We verified the validity of LSS scores by comparing them with manual coding results by human annotators. For this task, we used a subset of random public tweets (N=800) previously collected in a pilot study in which we constructed a large corpus of public tweets. Using the seed words listed in Table 1, we first calculated LSS scores using the pilot data, which is similar to the data set in this study. We asked 3 human annotators who are native Japanese speakers to independently read 800 tweets and, then, for each of them, judge the emotional status of the individual who tweeted it solely based on its content. In other words, the coders were only allowed to look at a single tweet, not the subsequent or previous posts by the same user or user profiles. Each of the 3 coders independently coded the 800 tweets as having a “good,” “neutral,” or “bad” emotional status. We gave each tweet a score of 2 (or –2) if the 3 coders rated it as “bad” (or “good”) and 1 (or –1) if the coding was split but 2 coders rated it as “bad” (or “good”). We assigned all other outcomes a score of 0. We then calculated the mean LSS score for each of the 5 human-annotated score categories (–2, –1, 0, 1, and 2) to examine their associations.

### Regression Analysis

To track weekly changes in the emotional distress level during the pandemic, we estimated the following model using individual tweets:



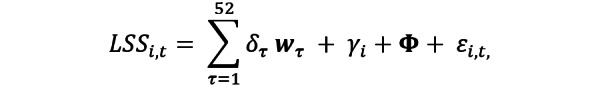



where *LSS_i,t_* is the *LSS* score of a tweet by individual *i* at time *t*, *w_τ_* is an indicator variable for week in the year 2020, *δ_τ_* is a coefficient associated with the weekly indicator *>w_τ_*, *γ_i_* is a fixed effect for individual *i*, and contains fixed effects for the week of the year and day of the week. Thus, the model considers the baseline heterogeneity across individuals, such as their inherent distress level and mental health conditions, as well as any baseline variations in the study participants’ mood over different periods, including seasonality (weekly variations) and within-week variations. We estimated the model for all the participants and then separately by their attribute (eg, sex and loneliness) to examine whether major events during the pandemic had varying effects on different groups of individuals. Note that the user-fixed effects were included in the model, and thus, no demographic controls were necessary and the estimation strategy exploited temporal variations in the LSS scores within participants. The SEs were clustered by participant.

## Results

### Sample Characteristics

Table 2 reports the number of participants by their attributes and levels of depressive symptoms, suicidal ideation, and loneliness. When we stratified the data by sex, we excluded 3 participants who selected “other” or “prefer not to answer” due to their small number. Similarly, those who chose “other” as their occupation (N=13, 2.3%) were excluded from the analysis when we conducted our analysis by employment status/type.

**Table 2:** Sample characteristics (N=560).

Characteristics			Participants, n (%)
**Sex (N=557)^a^**			
	Male	237 (42.5)	
	Female	320 (57.5)	
**Age (years)**			
	18-29	83 (14.8)	
	30-39	228 (40.7)	
	40-49	249 (44.5)	
**Income (JPY/US $)^b^**			
	<6 million (<44,551.20)	350 (62.5)	
	≥6 million (≥44,551.20)	210 (37.5)	
**Employment (N=547)^c^**			
	Part-time/temporary worker or unemployed	174 (31.8)	
	Permanent employee	294 (53.8)	
	Not in the labor force	79 (14.4)	
**Depressive symptoms**			
	No	384 (68.6)	
	Yes (PHQ-9^d^>=10)	176 (31.4)	
**Suicidal ideation^e^**			
	No	391 (69.8)	
	Yes	169 (30.2)	
**Loneliness**			
	Not lonely (3-item loneliness<6)	284 (50.7)	
	Lonely (3-item loneliness≥6)	276 (49.3)	

^a^Excluded 3 participants.

^b^JPY 1=US $ 0.0074.

^c^Excluded 13 participants.

^d^PHQ-9: 9-item Patient Health Questionnaire.

^e^Participants were categorized as “Yes” for the suicidal ideation question if they answered that they were bothered by thoughts that they would be better off dead or of hurting themselves in some way several days or more during the past 2 weeks in item 9 of the PHQ-9.

### LSS

#### Validation of LSS Scores

**Figure 1:**
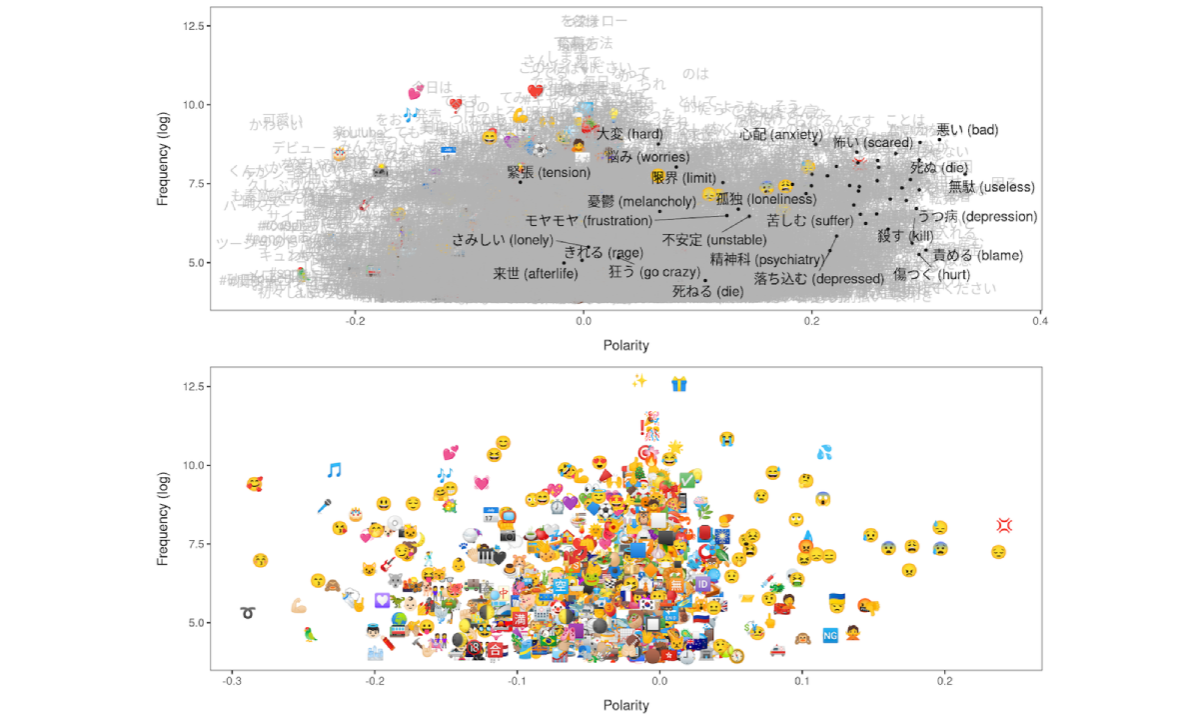
Estimated polarity of words and emojis. The x axis shows the estimated polarity score of words (top) and emojis (bottom) included in the data set, with higher values representing higher levels of emotional distress. The highlighted words in the top panel represent words that frequently appear in a crisis chat service, thus indicating a negative mental status.

First, we checked the overall validity of the LSS scores by visually inspecting the polarity scores assigned to words and emojis. In Figure 1, the first plot presents the estimated polarity of words and emojis on its x axis, with higher values indicating more negative states (ie, higher levels of distress). Their frequency in the corpus is shown on the y axis. The words highlighted in black represent words that frequently appear in a crisis chat service and, thus, those that are likely to be associated with high levels of emotional distress. The second plot shows the polarity score of only emojis. Both plots show that the words and emojis that represent negative emotions, topics, and issues received polarity scores greater than 0, which indicates higher distress levels.

**Figure 2:**
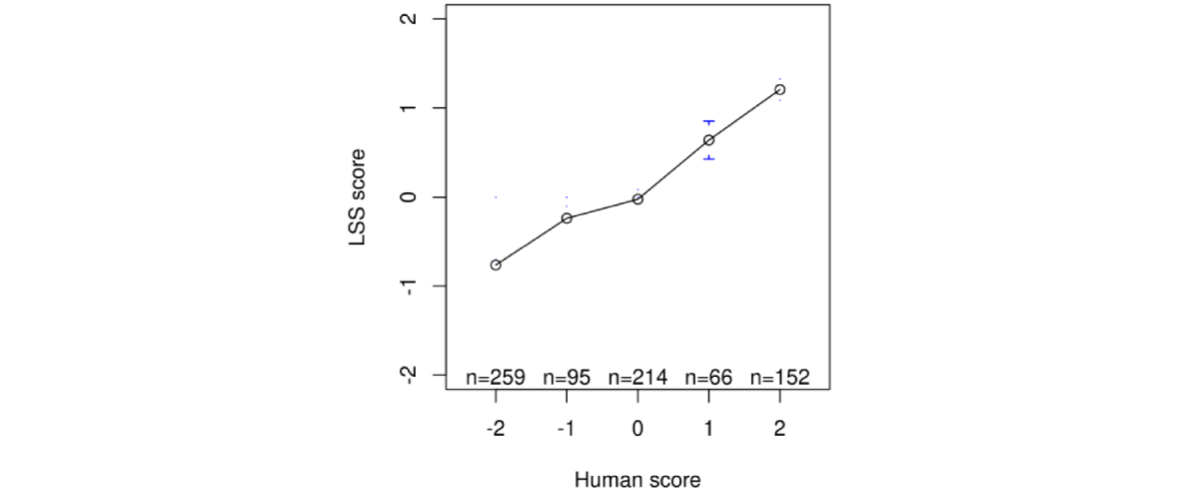
Mean LSS score vs human-annotated score. LSS: latent semantic scaling.

Second, we examined whether the LSS scores agreed with the annotation results by the 3 human coders. Figure 2 shows the mean LSS scores with 95% CIs for each level of human-annotated score. It shows that tweets that the human coders rated as exhibiting a “bad” mental state received higher polarity scores, suggesting that the LSS scores maintain a high level of consistency with ratings by human coders. The correlation between the LSS scores and human scores was r=0.71 (Pearson correlation coefficient).

#### Overview of LSS Scores

The mean LSS score during the study period was 0.092 (SD=1.030). The maximum and minimum scores were –5.888 and 5.050, respectively. Figure S1 in Multimedia Appendix 1 shows the distribution of the LSS scores. The number of tweets used in the analysis was 232,722 (47.66%) in 2019 and 255,562 (52.34%) in 2020. The descriptive statistics of LSS scores by user attribute and other characteristics are shown in Tables S1 and S2 in Multimedia Appendix 1. In addition, the weekly counts of tweets in 2020 are presented in Figure S2 in Multimedia Appendix 1, along with the overall distress level, which does not seem to be associated with the volume of tweets.

**Figure 3:**
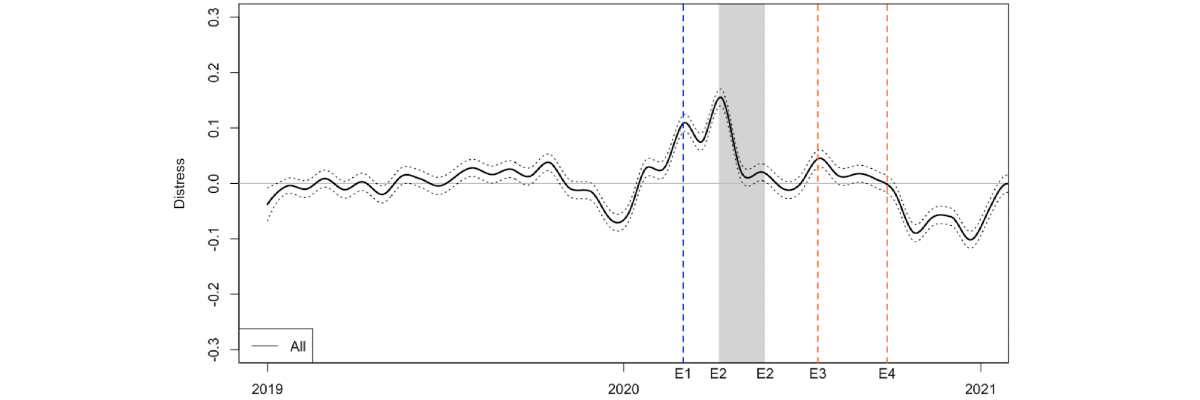
Trajectories of emotional distress: January 2019-December 2020. The blue vertical line (E1) denotes the week when school closure was announced and started, and the shaded area (E2) represents the period in which the state of emergency was in effect. The orange vertical lines (E3, E4) denote the weeks when the death by suicide of an entertainer was reported.

Figure 3 depicts the LSS scores to show how the overall distress level of the participants evolved during our study period. The LSS scores used in this figure are deviations from each participant’s mean score in 2019, and thus, the figure shows the average changes in the level of emotional distress from the pre–COVID-19 period. We smoothed the LSS scores by fitting the local regression model on the rolling 5% of all the data points. The figure shows the mean scores (solid line) with 95% CI bands (dotted lines). The blue dotted vertical line indicates the day school closure started (March 2, 2020) following the prime minister’s announcement 4 days earlier. The shaded area represents the period in which the first state of emergency was in effect (April 7-May 25, 2020). The vertical dotted orange lines indicate 2 instances when deaths by suicide of entertainers were reported (July 18 and September 27, 2020).

Figure 3 shows that the LSS score increased around the time of school closure in the initial phase of the pandemic in 2020, suggesting an elevated level of emotional distress. The overall LSS score elevated even further when a state of emergency was declared in early April 2020, but the level of emotional distress returned to the baseline period by the time the state of emergency was lifted. The overall LSS scores became negative by the fall of 2020, suggesting that the overall emotional distress level had decreased by then. There was no apparent increase in emotional distress levels around the time of the media reporting on celebrity suicides, although there was a minor increase around the time of the first instance.

**Figure 4:**
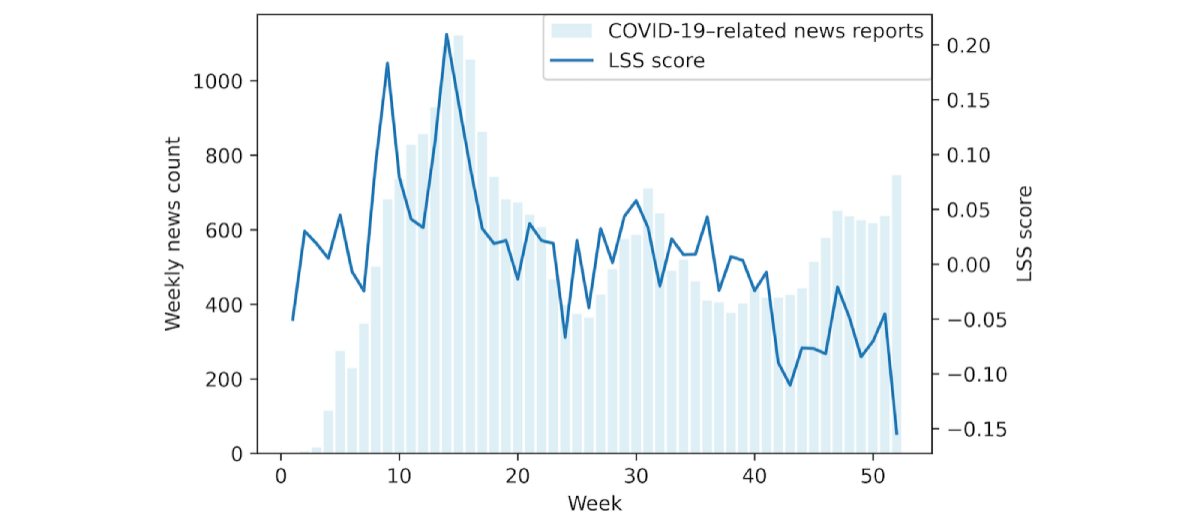
Number of COVID-19–related news reports and LSS scores in 2020. LSS: latent semantic scaling.

To examine the association between COVID-19–related news reports and the emotional distress level of Twitter users, Figure 4 illustrates the weekly total number of news reports and the weekly average LSS scores in 2020. Figure 4 indicates that the level of emotional distress increased when there was a rise in media reports on COVID-19 in the spring of 2020. By late June 2020 (week 26), the number of news reports on COVID-19 had decreased to its lowest level, and correspondingly, the emotional distress level, as indicated by LSS scores, also improved. Note that the news shown in the figure also contains positive news, including the development of messenger RNA (mRNA) vaccines and their approval for emergency use in December 2020, which might explain some discrepancies between the number of news reports and the LSS scores at the end of the year. As shown in Figure S3 in Multimedia Appendix 1, the number of confirmed cases does not seem to be associated with the estimated distress level.

### Regression-Based Analysis

**Figure 5:**
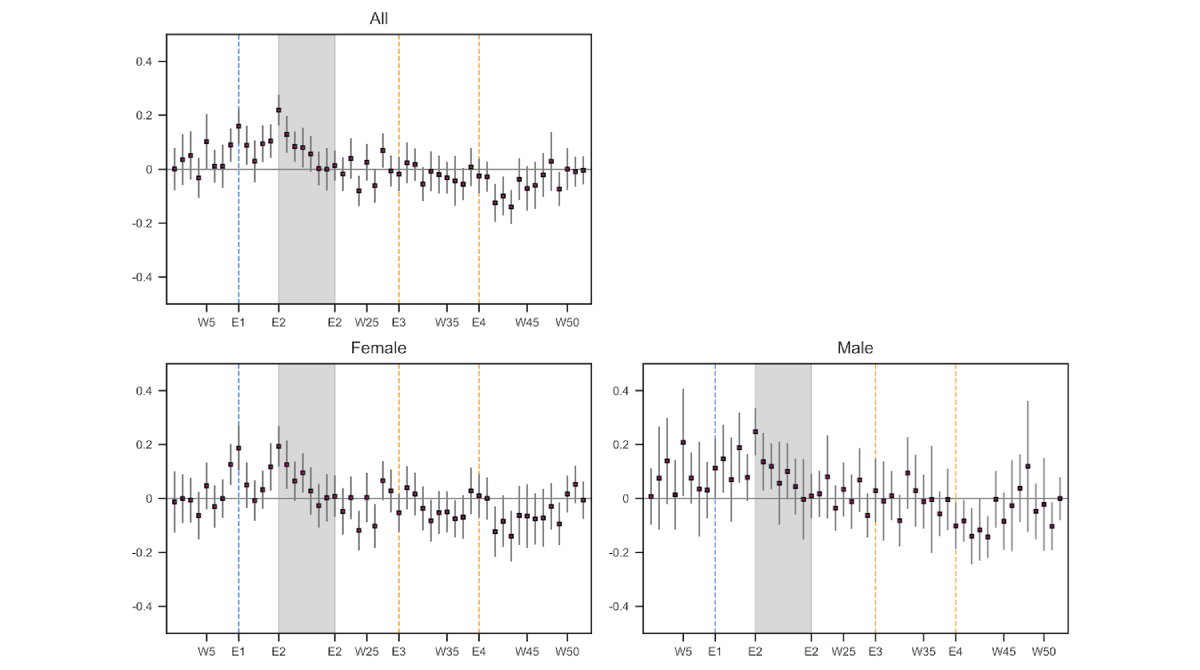
Estimated emotional distress levels in 2020 relative to 2019: all participants and by sex. The estimated models include user-fixed effects and week-of-year and day-of-week fixed effects. The blue vertical line (E1) denotes the week when school closure was announced and started, and the shaded area (E2) represents the period in which the state of emergency was in effect. The orange vertical lines (E3, E4) denote the weeks when the death by suicide of an entertainer was reported. The number of tweets used in the estimation was 488,284 (all), 292,002 (59.80%, female), and 189,562 (38.82%, male). The number of respondents in each category was 560 (all), 320 (57.14%, female), and 237 (42.32%, male). W: week.

Figure 5 shows the estimated coefficients for the weekly indicators in 2020 (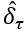 ) for all social media users and by sex, along with 95% CIs. Note that the coefficients shown are deviations from the corresponding baseline weeks and that user-specific characteristics were controlled for in estimating these coefficients. As in Figure 3, the blue dotted line (E1) denotes the week that school closure was announced and started (week 9, February 26-March 3), and the shaded areas indicate the period when the state of emergency was in place (E2, weeks 14-21). The orange dotted lines represent 2 instances in which celebrity suicides were reported (E3 and E4, weeks 29 and 39). The estimated coefficients are graphically presented in the main text, and their numerical values and *P* values are reported in Tables S3-S10 in Multimedia Appendix 1. Table S3 also lists the dates for each week and a brief description of major COVID-19–related events.

The top panel of Figure 5 confirms the general pattern observed in Figure 3; the level of emotional distress increased in the week when school closure started, and it peaked at the beginning of the state of emergency (E2, week 14). The estimated coefficient for week 14 was 0.219 (95% CI 0.162-0.276). At the same time, there was no statistically significant change in the emotional distress levels in weeks when unexpected deaths by suicide of entertainers were reported (E3 and E4), once user attributes and baseline temporal fluctuations in distress levels were controlled for.

According to the bottom panel of Figure 5, there was a statistically significant elevation in the level of emotional distress among females when school closure started (E1; estimated coefficient=0.187, 95% CI 0.104-0.271), but the magnitude of the change among males was smaller (estimated coefficient=0.113, 95% CI 0.001-0.224). Both males and females seemed to have experienced a larger increase in emotional distress at the beginning of the state of emergency, but the distress levels of both groups returned to the baseline in about a month.

**Figure 6:**
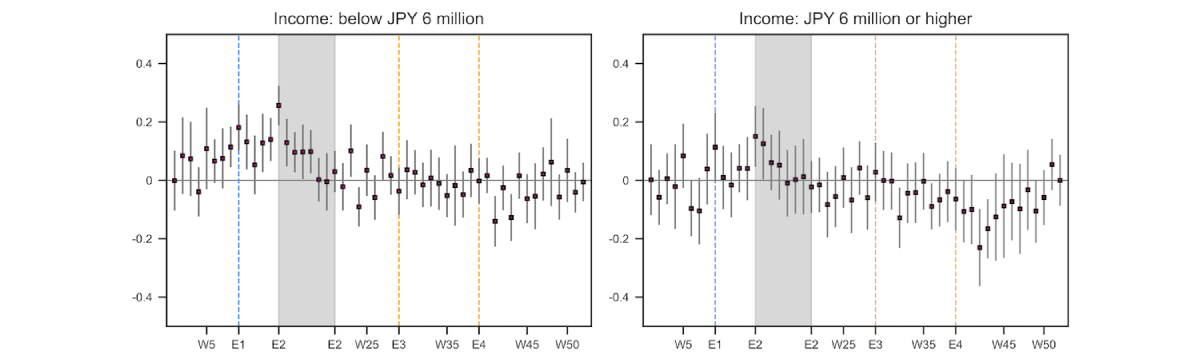
Estimated emotional distress level in 2020 relative to 2019: by household income. The number of tweets used in the estimation was 317,767 (less than JPY 6 million/US $ 44,551.20) and 170,517 (equal to or more than JPY 6 million). The number of respondents in each category was 350 (less than JPY 6 million) and 210 (equal to or more than JPY 6 million). The blue vertical line (E1) denotes the week when school closure was announced and started, and the shaded area (E2) represents the period in which the state of emergency was in effect. The orange vertical lines (E3, E4) denoted the weeks when the death by suicide of an entertainer was reported. W: week.

We also estimated the model separately by age group, income, and employment status/type of job. We found that those in the youngest age group (18-29 years old) experienced the largest increase around the time of school closure (E1, Figure S4 in Multimedia Appendix 1). In addition, the youngest group did not experience an improvement in distress levels in the fall and winter of 2020. In contrast, the distress level of those in their forties became even lower than in 2019 during the last several months of 2020. As shown in Figure 6, the analysis by income group indicated that there was a statistically significant increase in the level of emotional distress among the low-income group since week 8, even before school closure was announced and started in week 9 (E1), until week 18 (except for week 11, when their distress level was indistinguishable from that in 2019). Individuals with a relatively higher income experienced only a brief increase in their distress level at the outset of the state of emergency (E2).

**Figure 7:**
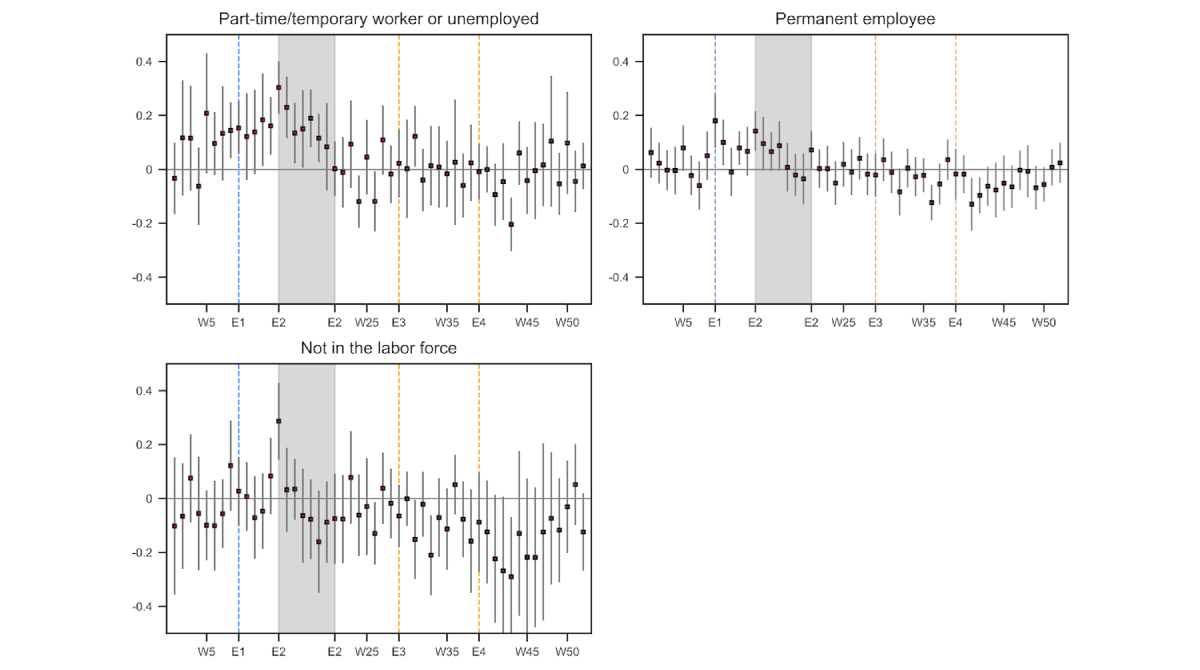
Estimated emotional distress level in 2020 relative to 2019: by type of employment and employment status. The number of tweets used in the estimation was 170,269 (part-time/temporary worker or unemployed), 238,218 (permanent employee), and 68,215 (not in the labor force). The number of respondents in each category was 174 (part-time/temporary worker or unemployed), 294 (permanent employee), and 79 (not in the labor force). The blue vertical line (E1) denotes the week when school closure was announced and started, and the shaded area (E2) represents the period in which the state of emergency was in effect. The orange vertical lines (E3, E4) denoted the weeks when the death by suicide of an entertainer was reported. W: week.

Figure 7 shows the regression results when the participants were categorized by the type and status of employment. Those with an unstable job or those who were unemployed experienced a statistically significant increase in their emotional distress level for 8 weeks in the spring of 2020 (weeks 12-19), even before the declaration of the state of emergency (E2), whereas such a prolonged elevation in distress levels was not observed among those with a stable job or those not in the labor force.

**Figure 8:**
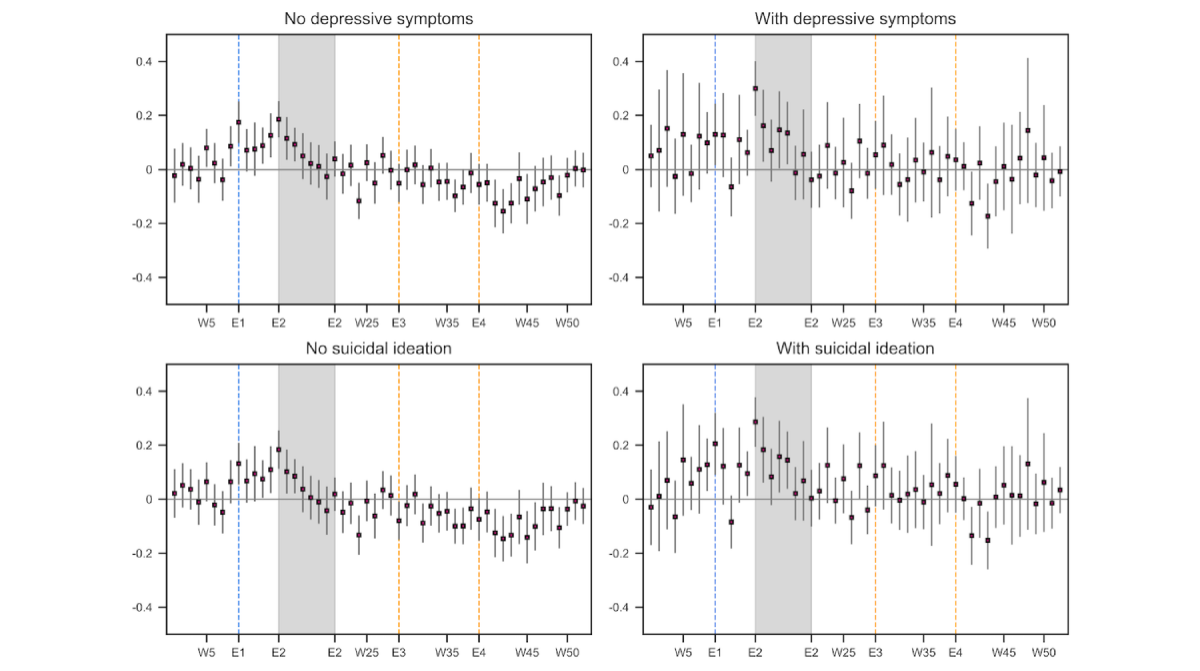
Estimated emotional distress level in 2020 relative to 2019: by the presence of depressive symptoms and suicidal ideation. The number of tweets used in the estimation was 331,227 (no depressive symptoms), 157,057 (with depressive symptoms), 307,018 (no suicidal ideation), and 181,266 (with suicidal ideation). The number of respondents in each category was 384 (no depressive symptoms), 176 (with depressive symptoms), 391 (no suicidal ideation), and 169 (with suicidal ideation). The blue vertical line (E1) denotes the week when school closure was announced and started, and the shaded area (E2) represents the period in which the state of emergency was in effect. The orange vertical lines (E3, E4) denoted the weeks when the death by suicide of an entertainer was reported. W: week.

The regression results by depressive symptoms and suicidality (Figure 8) indicated that those with no depressive symptoms experienced a rise in their distress level at the outset of the state of emergency (E2), but their distress level returned to the baseline level by week 4 into the emergency declaration. In contrast, those with depressive symptoms experienced a larger and more prolonged rise in their distress level during the state of emergency. However, once the state of emergency was over, their distress level returned to the baseline period.

A similar picture emerged from the analysis by the level of suicidality (bottom panel, Figure 8). Those who had occasional or more frequent suicidal ideation experienced a longer, heightened level of emotional distress during the state of emergency, but such a pattern was not observed among nonsuicidal individuals. We did not observe any statistically significant increase in the level of emotional distress, even among those with depressive symptoms or suicidal ideations, in the weeks when celebrity suicides were reported (E3 and E4).

Finally, individuals who were classified as lonely experienced a larger and longer increase in their emotional distress level during the state of emergency compared to their nonlonely counterparts, but their level of distress returned to the baseline level by the end of the state of emergency (Figure S5 in Multimedia Appendix 1). Aside from the rise in distress levels during the state of emergency, their pattern of emotional distress did not differ much from those who were not lonely.

## Discussion

### Principal Findings

This study proposed a machine learning framework for the real-time surveillance of the mental health conditions of social media users that does not require extensive training data. Using a semisupervised algorithm and survey-linked tweets, the study estimated the level of emotional distress among a subset of Twitter users in Japan during the COVID-19 pandemic. We validated LSS scores by visually inspecting the estimated polarity of words and emojis in the corpus and by confirming their correlation with human-annotated results. Having confirmed the validity of the LSS scores, we then observed the trajectories of the overall LSS scores in 2019 and 2020. We found that the emotional distress level of the study participants was elevated when major social restrictions were introduced or when the number of news reports on COVID-19 increased. We also found that the level of emotional distress of our study participants was unrelated to the number of positive COVID-19 cases. These results suggest that containment policies, not the spread of the disease itself or associated fears, were a source of emotional distress for these social media users during the early months of the pandemic. This finding is consistent with suicide-related call volumes received by crisis chat or phone hotline services in other countries that were positively correlated with the introduction of containment policies rather than with infection rates [28,29].

Our regression analysis revealed precise timings when our participants’ emotional distress elevated relative to the prepandemic level. Such precise measurements would not be possible without the high-frequency data in this study. For our study participants, the two COVID-19–related events associated with the elevated emotional distress levels were school closure in early March and the declaration of a state of emergency in April 2020. Our results also suggest that school closure may have had a slightly larger impact on females than males. School closure might have disproportionately affected Japanese working females because they tend to bear a greater burden of childcare and domestic work responsibilities than those in other high-income countries [30]. The number of female workers who took a leave of absence in March-May 2020, when school closure was in effect, was 7.7 million, whereas 3.5 million male counterparts took leave from work during the same period [31]. Further, the impact of school closure on employment status is estimated to be larger among female workers with a spouse and young children than among female workers with a spouse but no child [32].

More importantly, our analysis clearly revealed socioeconomic attributes associated with higher levels of distress during the pandemic. We found that the study participants with low income suffered more heavily and persistently than those with relatively higher incomes. Note that our analysis adjusted for the underlying socioeconomic conditions of each individual. We also found that school closure and the state of emergency asymmetrically affected permanent and nonpermanent workers in our sample. The abrupt closure of schools and childcare facilities and the closure of shops and restaurants during the state of emergency left many part-time and hourly workers out of work, with no or little wage compensation. Some even lost their jobs; the number of female workers with nonpermanent positions decreased by 0.7 million in April 2020 alone compared to the same month in 2019, and the number kept declining throughout 2020, while the number of permanent workers continued its increasing trend since the prepandemic period [32].

Throughout the analysis, we did not find any evidence that the media reporting on celebrities’ suicides affected the emotional distress levels of our study participants, including those with depressive symptoms and suicidal ideation. The result is somewhat unexpected, given the presence of numerous studies demonstrating that the number of deaths by suicide tends to increase following the media reporting of prominent suicides [10,11]. Moreover, the repercussions of such media reporting have been detected among Twitter users as well; they tend to react emotionally in response to media reports on prominent deaths by suicide, especially when the deceased is a relatively young entertainer and when the death is regarded as unexpected [12], which exactly matches the descriptions of the 2 entertainers who killed themselves in 2020. In fact, the number of deaths by suicide in Japan suddenly increased in July 2020, when the media reported the first incident, although the number of deaths by suicide was lower than the previous trend in the first several months of the pandemic. In October 2020, right after the media reporting on the second prominent suicide, the number of female suicides exhibited a sharp spike, with about a 70% increase compared to the same month in the previous 5 years [33]. One potential reason that our study did not detect any elevation in the emotional distress level following the media reports might lie in our seed words; they were constructed to capture the general emotional distress level, which can be distinct from suicidal ideation. Another reason might be related to the fact that deaths by suicide are rare; even in the month that recorded the highest number of deaths by suicide in 2020 (October), the number of deaths by suicide was 2230 [34], which amounts to 1.776 deaths per 100,000 persons in Japan. It is possible that a much larger sample is needed to capture a change in the level of emotional distress of those who might be significantly affected by the media reporting of celebrity suicides.

### Limitations

There are several limitations of this study. First, our results are based on a relatively small number of Twitter users and, thus, cannot be generaliazable to the general population or even all the social media users in Japan. Thus, caution is warranted when interpreting our substantive findings. Second, as with all other studies that have analyzed social media data, our findings may not be generalizable beyond social media users. Even though Twitter is one of the popular social media platforms in Japan, its users may not necessarily be representative of the general public. In addition, our sampling scheme was not designed to create a representative sample of the general population or Twitter users in Japan. Third, as noted earlier, our classifications of the participants may not necessarily reflect their attributes or conditions during the peak period of the pandemic or the baseline period. We measured the participants’ attributes and conditions in May 2022, and our assumption that they had remained largely unchanged since 2020 may not hold true. For example, individuals who were classified as “suicidal” in our study based on their PHQ-9 score in 2022 may have been in the “not suicidal” group in 2020, but we are unable to check this possibility. Fourth, it is possible that individuals suffering from adverse mental health conditions are more likely to use a private account, which they can maintain separately from their public account, to reveal their true feelings and conditions, and thus, our study may have underestimated their emotional distress levels. Thus, caution is warranted in interpreting the results, especially regarding those related to at-risk individuals. Finally, we restrospectively retrieved past tweets, and thus, our data set lacks tweets that were deleted prior to our data retrival in May 2022. In particular, it is possible that some of our participants deleted their past tweets about their distressed conditions during the peak period of the pandemic, which might have resulted in the underestimation of their distress level.

### Strengths and Contributions

Nevertheless, this study makes several important contributions both substantively and methodologically. Substantively, to the best of our knowledge, our study constitutes the first to track the trajectories of emotional distress levels of social media users, separately by user attributes. Previous studies could only understand the sentiments of overall Twitter users [2-8] or had to rely on imputed demographic data [35]. Moreover, no previous studies have analyzed the contents of tweets by the psychological conditions of users, such as the presence of depressive symptoms, suicidal ideation, and loneliness, using the standard constructs of these conditions. Another strength of our study is that we were able to estimate the emotional distress level of social media users during the pandemic and in the prepandemic period. Thus, we could detect changes during the pandemic period relative to their underlying emotional distress levels.

This study also makes a significant methodological contribution to the field by establishing a framework to implement near-real-time monitoring of the emotional distress level of social media users. LSS is a semisupervised algorithm that requires only a few seed words for training. Large annotated data sets for mental health conditions are rarely available; thus, supervised learning algorithms are often infeasible or too costly. Even if such data sets existed, annotated data sets could become quickly obsolete, especially in the social media domain, where new usage of words, acronyms, initialisms, and emoticons constantly emerge and disappear. LSS is also advantageous over lexicon- or rule-based sentiment analysis methods, such as Linguistic Inquiry and Word Count (LIWC) [36] and Valence Aware Dictionary and Sentiment Reasoner (VADER) [37] because its semisupervised algorithm can identify domain-specific polarity words in the corpus.

Given its flexibility and adaptability, our framework is easily extendable for other purposes. For example, it is straightforward to apply our framework to measure the degree of suicidality or loneliness once we identify appropriate seed words that characterize these conditions. Moreover, performing the same analysis on a corpus of tweets in other languages (eg, English, German, Chinese, Arabic) only requires translating our seed words.

Thus, the framework proposed in this study can have multiple applications. It can be used on streaming data for continuous measurement of the conditions and sentiments of any group of interest, as long as the text data written by the targeted group are available. The text data that can benefit from the proposed framework include comments in discussion forums (eg, Reddit) and social media posts, and online conversations, such as crisis text services. Another application of our framework includes detecting distressed individuals on social media, to whom tailored messages may be sent to promote their help-seeking behavior.

### Conclusion

Our study highlights a great potential to continuously monitor the psychological health of social media users with the proposed method as a complement to administrative and large-scale survey data. Our findings also suggest that the government-induced events in the early phase of the COVID-19 pandemic disproportionately affected the psychological conditions of vulnerable individuals, including those with a low income, precarious employment, depressive symptoms, and suicidal ideation.

## Appendix

Multimedia Appendix 1 Supplementary tables and figures.

## Abbreviations

API: application programming interface
LSS: latent semantic scaling
PHQ-9: 9-item Patient Health Questionnaire
SVD: singular vector decomposition

## Data Availablity

The study’s data contain potentially identifying information about Twitter users and thus cannot be shared. They can only be accessed by members of the research team, as per participant consent and the contract with the survey company. The analysis script is available from the corresponding author upon request.
