# Correction: Emotional Distress During COVID-19 by Mental Health Conditions and Economic Vulnerability: Retrospective Analysis of Survey-Linked Twitter Data With a Semisupervised Machine Learning Algorithm

**DOI:** 10.2196/47549

**Published:** 2023-03-27

**Authors:** Michiko Ueda, Kohei Watanabe, Hajime Sueki

**Affiliations:** 1 Department of Public Administration and International Affairs The Maxwell School of Citizenship and Public Affairs Syracuse University Syracuse, NY United States; 2 Center for Policy Research The Maxwell School of Citizenship and Public Affairs Syracuse University Syracuse, NY United States; 3 Waseda Institute for Advanced Study Waseda University Tokyo Japan; 4 Faculty of Human Sciences Wako University Tokyo Japan

In “Emotional Distress During COVID-19 due to Mental Health Conditions and Economic Vulnerability: Retrospective Analysis of Survey-Linked Twitter Data With a Semisupervised Machine Learning Algorithm” (J Med Internet Res 2023;25:e44965), the authors noted one error.

The title “Emotional Distress During COVID-19 due to Mental Health Conditions and Economic Vulnerability: Retrospective Analysis of Survey-Linked Twitter Data With a Semisupervised Machine Learning Algorithm” has been changed to “Emotional Distress During COVID-19 by Mental Health Conditions and Economic Vulnerability: Retrospective Analysis of Survey-Linked Twitter Data With a Semisupervised Machine Learning Algorithm.”

The correction will appear in the online version of the paper on the JMIR Publications website on March 27, 2023, together with the publication of this correction notice. Because this was made after submission to PubMed, PubMed Central, and other full-text repositories, the corrected article has also been resubmitted to those repositories.

