# Supplementary material for: Emotional Distress During COVID-19 by Mental Health Conditions and Economic Vulnerability: Retrospective Analysis of Survey-Linked Twitter Data With a Semisupervised Machine Learning Algorithm
Source: J Med Internet Res. 2023 Mar 16;25:e44965. doi: 10.2196/44965 (PMC10022650; doi:10.2196/44965)
Supplement: Multimedia Appendix 1 [file jmir_v25i1e44965_app1.pdf]

Multimedia Appendix for Emotional distress during COVID-19 by mental health conditions and economic vulnerability: Retrospective analysis of survey-linked Twitter data with a semi-supervised machine learning algorithm

Michiko Ueda, Ph.D[1-2], Kohei Watanabe, Ph.D[3], Hajime Sueki, Ph.D[4]

1. Department of Public Administration and International Affairs, Syracuse University, Syracuse, New York, United States.
2. Center for Policy Research, Maxwell School of Citizenship and Public Affairs, Syracuse University, Syracuse, New York, United States.
3. Waseda Institute for Advanced Study, Waseda University, Tokyo, Japan.
4. Faculty of Human Sciences, Wako University, Tokyo, Japan.

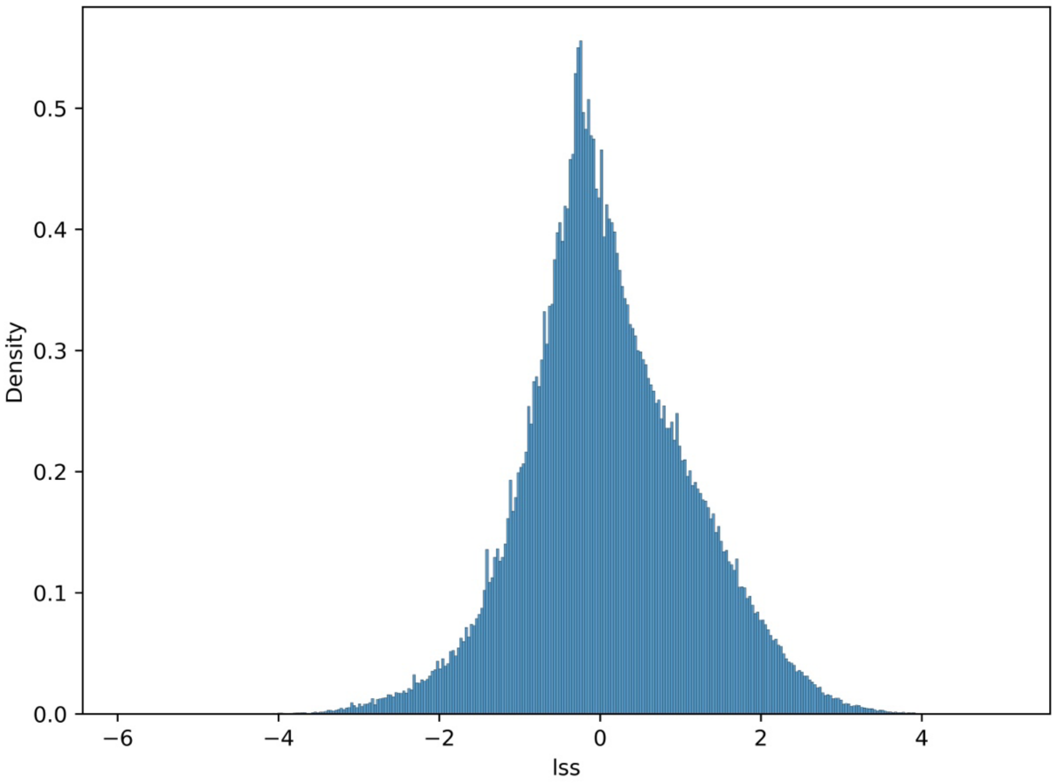

Figure S1. the distribution of LSS scores.

Note: N=488,284. LSS scores represent estimated emotional distress level.

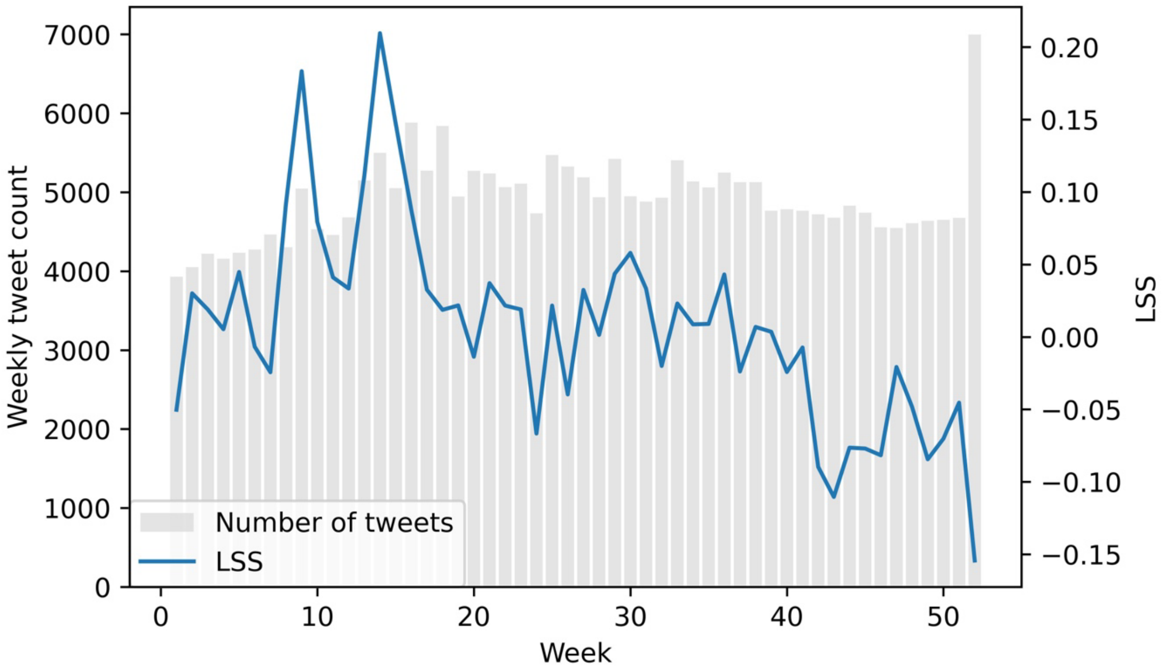

Figure S2. Weekly Tweet counts and LSS in 2020.

Note: LSS scores represent estimated emotional distress level with higher values indicate higher distress level.

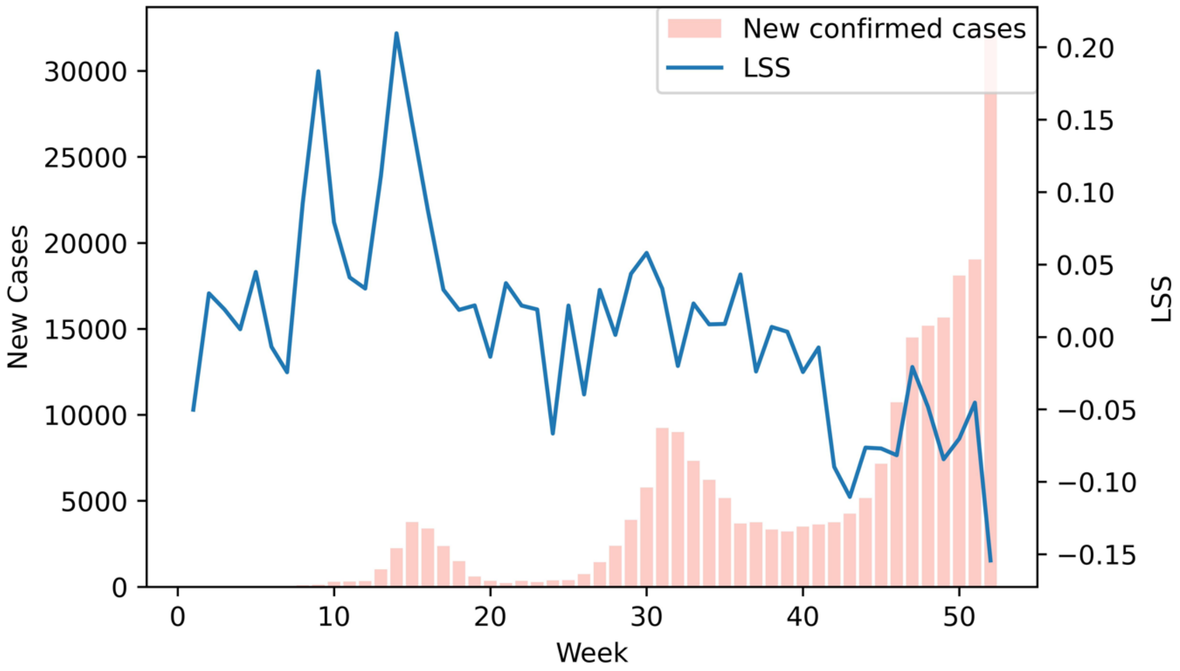

Figure S3. The number of new confirmed COVID cases in Japan and LSS in 2020.

Note: LSS scores represent estimated emotional distress level with higher values indicate higher distress level.

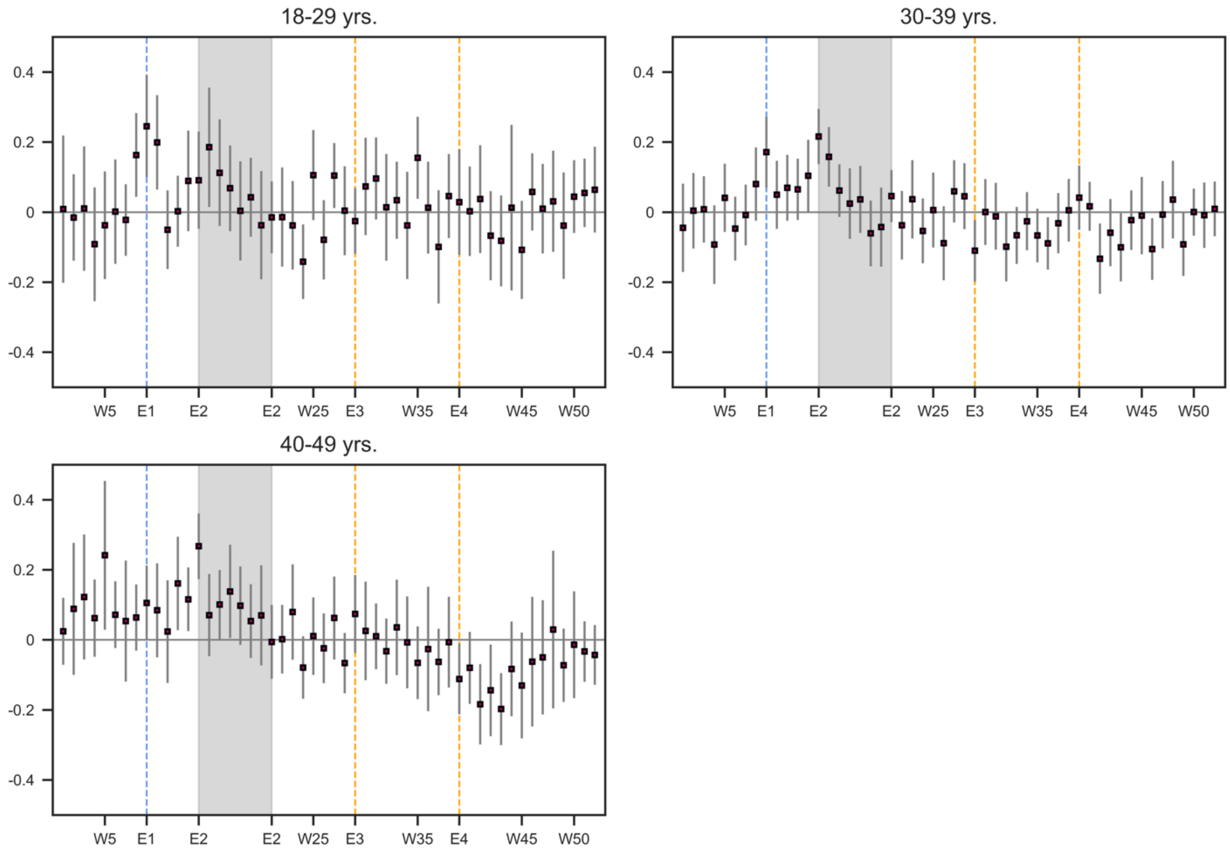

Figure S4. Estimated emotional distress level in 2020: by age group.

Note: The estimated coefficients on weekly indicators in 2020 with 95% confidence intervals are shown. The dependent variable is estimated emotional distress level (LSS score). The estimated models include user-fixed effects and week-of-year and day-of-week fixed effects. The blue vertical line (E1) denotes the week when school closure was announced and started, and the shaded area (E2) represents the period in which the state of emergency was in effect. The orange vertical lines (E3, E4) denoted the weeks when the suicide death of an entertainer was reported. W: week.

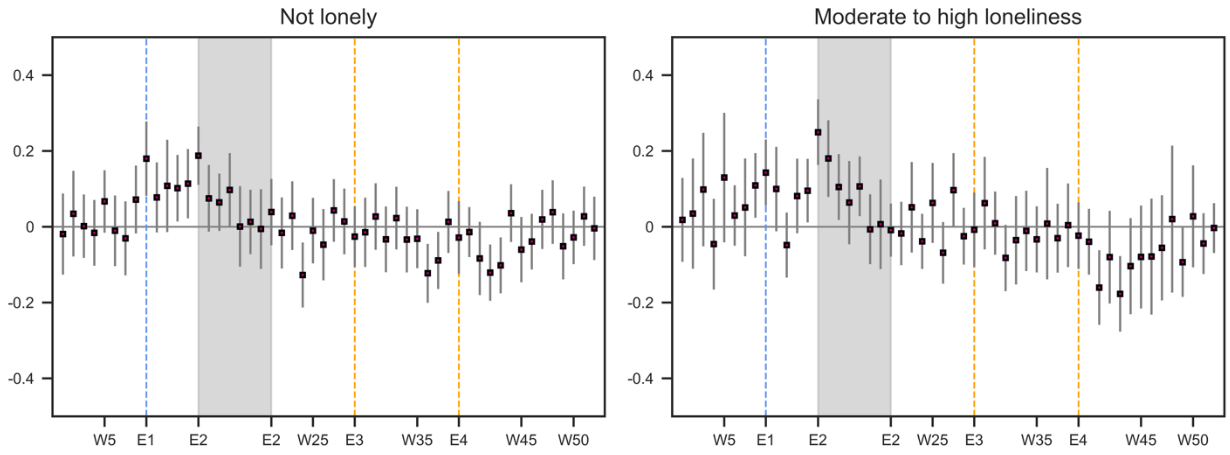

Figure S5. Estimated emotional distress level in 2020: by loneliness.

Note: The estimated coefficients on weekly indicators in 2020 with 95% confidence intervals are shown. The dependent variable is estimated emotional distress level (LSS score). The estimated models include user-fixed effects and week-of-year and day-of-week fixed effects. The blue vertical line (E1) denotes the week when school closure was announced and started, and the shaded area (E2) represents the period in which the state of emergency was in effect. The orange vertical lines (E3, E4) denoted the weeks when the suicide death of an entertainer was reported. W: week.

Table S1: LSS scores by participants' attribute/condition

		count	mean	std	min	50%	max
All		488284	0.092	1.031	-5.888	-0.005	5.050
Sex	Male	189562	0.182	1.028	-4.803	0.061	4.618
	Female	292002	0.030	1.031	-5.888	-0.054	5.050
Age	18-29 yrs	81883	0.159	1.000	-4.116	0.081	5.046
	30-39 yrs	200424	0.084	1.017	-5.888	-0.012	5.050
	40-49 yrs	205977	0.072	1.055	-5.001	-0.035	4.618
Income	<6 million yen	317767	0.128	1.030	-5.888	0.034	5.050
	6>= million yen	170517	0.025	1.030	-5.165	-0.075	4.850
Employment	Part-time/temporary worker or unemployed	170269	0.100	1.076	-5.888	0.005	5.050
	Permanent Employee	238218	0.100	0.986	-4.706	0.008	4.850
	Not in the labor force	68215	0.024	1.079	-5.095	-0.077	5.016
Depressive symptoms	No	331227	0.095	1.026	-5.888	-0.010	4.776
	Yes (PHQ-9>=10)	157057	0.085	1.041	-5.165	0.006	5.050
Suicidal ideation	No	307018	0.089	1.016	-5.888	-0.014	5.050
	Yes (several days or more in 2 wks)	181266	0.096	1.056	-5.165	0.011	5.046
Loneliness	Low (3-item loneliness<6)	232957	0.059	1.008	-5.888	-0.035	5.050
	Moderate to high (3-item loneliness>=6)	255327	0.122	1.050	-5.165	0.022	5.046

Table S2: LSS by participants' attribute/condition and year

		2019						2020					
		count	mean	std	min	50%	max	count	mean	std	min	50%	max
All		232722	0.090	1.020	-4.817	0.005	5.050	255562	0.093	1.041	-5.888	-0.015	5.046
Sex	Male	88899	0.171	1.026	-4.004	0.067	4.510	100663	0.191	1.029	-4.803	0.054	4.618
	Female	139954	0.036	1.015	-4.817	-0.043	5.050	152048	0.025	1.046	-5.888	-0.067	5.046
Age	18-29 yrs	43763	0.153	0.984	-4.116	0.076	4.675	38120	0.167	1.018	-4.021	0.087	5.046
	30-39 yrs	96736	0.087	1.017	-4.817	0.000	5.050	103688	0.081	1.017	-5.888	-0.022	4.789
	40-49 yrs	92223	0.064	1.037	-4.780	-0.025	4.442	113754	0.079	1.069	-5.001	-0.046	4.618
Income	<6 million yen	149274	0.131	1.020	-4.817	0.055	5.050	168493	0.125	1.038	-5.888	0.017	5.046
	6>= million yen	83448	0.018	1.015	-4.441	-0.070	4.510	87069	0.031	1.044	-5.165	-0.076	4.850
Employment	Part-time/temporary worker or unemployed	78551	0.094	1.062	-4.817	0.015	5.050	91718	0.105	1.088	-5.888	-0.006	5.046
	Permanent Employee	119093	0.090	0.978	-4.441	0.011	4.675	119125	0.110	0.994	-4.706	0.005	4.850
	Not in the labor force	29277	0.076	1.090	-4.361	-0.040	5.016	38938	-0.015	1.069	-5.095	-0.113	4.789
Depressive symptoms	No	158338	0.090	1.005	-4.817	-0.007	4.776	172889	0.100	1.045	-5.888	-0.012	4.618
	Yes (PHQ-9>=10)	74384	0.091	1.051	-4.361	0.022	5.050	82673	0.079	1.032	-5.165	-0.019	5.046
Suicidal ideation	No	144448	0.086	0.998	-4.817	-0.012	5.050	162570	0.091	1.032	-5.888	-0.015	4.789
	Yes (several days or more in 2 wks)	88274	0.097	1.055	-4.439	0.027	5.016	92992	0.096	1.056	-5.165	-0.014	5.046
Loneliness	Low (3-item loneliness<6)	112197	0.041	0.983	-4.817	-0.043	5.050	120760	0.075	1.031	-5.888	-0.028	4.789
	Moderate to high (3-item loneliness>=6)	120525	0.136	1.050	-4.439	0.047	5.016	134802	0.109	1.050	-5.165	-0.001	5.046

Table S3: Estimated emotional distress level in 2020: all participants.

Week	Dates	Major event	Coef	95% CI	p-value
1	1/1 - 1/7		0.001	[-0.078, 0.079]	(0.984)
2	1/8 - 1/14		0.035	[-0.059, 0.130]	(0.460)
3	1/15 - 1/21	First confirmed case in Japan (1/15)	0.051	[-0.039, 0.141]	(0.264)
4	1/22 - 1/28		-0.032	[-0.107, 0.042]	(0.396)
5	1/29 - 2/4	Confirmed cases on Diamond Princess	0.102	[-0.001, 0.205]	(0.051)
6	2/5 - 2/11		0.011	[-0.050, 0.072]	(0.721)
7	2/12 - 2/18		0.011	[-0.070, 0.092]	(0.788)
8	2/19 - 2/25		0.090	[0.027, 0.152]	(0.005)
9	2/26 - 3/3	School closure requested (2/27) and started (3/2)	0.159	[0.094, 0.225]	(0.000)
10	3/4 - 3/10		0.089	[0.016, 0.161]	(0.017)
11	3/11 - 3/17	WHO declared COVID "pandemic" (3/11)	0.030	[-0.048, 0.107]	(0.454)
12	3/18 - 3/24	Tokyo Summer Olympics postponed (3/24)	0.094	[0.026, 0.163]	(0.007)
13	3/25 - 3/31	Death of popular comedian by COVID reported (3/30)	0.104	[0.042, 0.167]	(0.001)
14	4/1 - 4/7	State of emergency declaration in urban areas (4/7)	0.219	[0.162, 0.276]	(0.000)
15	4/8 - 4/14		0.129	[0.061, 0.198]	(0.000)
16	4/15 - 4/21	Nationwide state of emergency declaration (4/16); one-time cash payment (about 750 USD/pp) announced	0.084	[0.028, 0.141]	(0.004)
17	4/22 - 4/28		0.080	[0.006, 0.154]	(0.033)
18	4/29 - 5/5	State of emergency extended (5/4)	0.057	[-0.009, 0.123]	(0.091)
19	5/6 - 5/12		0.003	[-0.060, 0.066]	(0.932)
20	5/13 - 5/19		-0.000	[-0.079, 0.079]	(0.995)
21	5/20 - 5/26	State of emergency lifted (5/25)	0.013	[-0.043, 0.069]	(0.642)
22	5/27 - 6/2	By 6/1, 90% of public school reopened	-0.018	[-0.080, 0.045]	(0.576)
23	6/3 - 6/9		0.040	[-0.035, 0.115]	(0.299)
24	6/10 - 6/16		-0.080	[-0.137, -0.024]	(0.006)
25	6/17 - 6/23	Travel restrictions across prefecture borders lifted (6/19	0.026	[-0.043, 0.094]	(0.460)
26	6/24 - 6/30		-0.061	[-0.124, 0.002]	(0.058)
27	7/1 - 7/7		0.069	[0.005, 0.134]	(0.034)
28	7/8 - 7/14		-0.006	[-0.064, 0.051]	(0.828)
29	7/15 - 7/21	Suicide death of 30-year-old actor reported (7/18)	-0.018	[-0.081, 0.045]	(0.576)
30	7/22 - 7/28	Domestic travel subsidy program (GO TO travel) startec	0.024	[-0.052, 0.100]	(0.539)
31	7/29 - 8/4		0.017	[-0.043, 0.078]	(0.572)
32	8/5 - 8/11		-0.055	[-0.118, 0.008]	(0.087)
33	8/12 - 8/18		-0.007	[-0.081, 0.067]	(0.846)
34	8/19 - 8/25		-0.020	[-0.091, 0.050]	(0.575)
35	8/26 - 9/1		-0.032	[-0.091, 0.027]	(0.293)
36	9/2 - 9/8		-0.043	[-0.136, 0.050]	(0.361)
37	9/9 - 9/15		-0.055	[-0.116, 0.005]	(0.071)
38	9/16 - 9/22		0.008	[-0.063, 0.078]	(0.824)
39	9/23 - 9/29	Suicide death of 40-year-old actress reported (9/27)	-0.025	[-0.089, 0.039]	(0.444)
40	9/30 - 10/6		-0.028	[-0.084, 0.028]	(0.327)
41	10/7 - 10/13		-0.125	[-0.195, -0.054]	(0.001)
42	10/14 - 10/20		-0.099	[-0.171, -0.027]	(0.007)
43	10/21 - 10/27		-0.140	[-0.203, -0.077]	(0.000)
44	10/28 - 11/3		-0.038	[-0.115, 0.040]	(0.343)
45	11/4 - 11/10		-0.071	[-0.154, 0.011]	(0.091)
46	11/11 - 11/17		-0.060	[-0.147, 0.027]	(0.177)
47	11/18 - 11/24		-0.021	[-0.103, 0.061]	(0.615)
48	11/25 - 12/1		0.029	[-0.080, 0.138]	(0.603)
49	12/2 - 12/8		-0.073	[-0.136, -0.010]	(0.022)
50	12/9 - 12/15		0.000	[-0.077, 0.078]	(0.996)
51	12/16 - 12/22		-0.009	[-0.065, 0.046]	(0.746)
52	12/23 - 12/31	Domestic travel subsidy program ended (12/8)	-0.004	[-0.057, 0.048]	(0.876)
N (Tweets)				488284	
N (users)				560	

Note: The estimated coefficients on weekly indicators in 2020 with 95% confidence intervals are shown. The estimated models include user-fixed effects and week-of-year and day-of-week fixed effects.

Table S4: Estimated emotional distress level in 2020: by sex.

	Female			Male		
Week	Coef	95% CI	p-value	Coef	95% CI	p-value
1	-0.013	[-0.127, 0.101]	(0.822)	0.007	[-0.097, 0.112]	(0.888)
2	-0.001	[-0.091, 0.090]	(0.991)	0.075	[-0.116, 0.266]	(0.439)
3	-0.006	[-0.089, 0.077]	(0.886)	0.139	[-0.021, 0.300]	(0.088)
4	-0.064	[-0.152, 0.025]	(0.157)	0.014	[-0.115, 0.143]	(0.833)
5	0.047	[-0.040, 0.134]	(0.292)	0.208	[0.009, 0.407]	(0.041)
6	-0.030	[-0.108, 0.048]	(0.453)	0.076	[-0.019, 0.170]	(0.116)
7	-0.000	[-0.072, 0.071]	(0.989)	0.035	[-0.141, 0.211]	(0.697)
8	0.126	[0.050, 0.202]	(0.001)	0.031	[-0.074, 0.136]	(0.557)
9	0.187	[0.104, 0.271]	(0.000)	0.113	[0.001, 0.224]	(0.047)
10	0.050	[-0.035, 0.134]	(0.246)	0.147	[0.021, 0.274]	(0.022)
11	-0.008	[-0.083, 0.068]	(0.844)	0.070	[-0.087, 0.226]	(0.380)
12	0.032	[-0.038, 0.103]	(0.367)	0.189	[0.058, 0.319]	(0.005)
13	0.117	[0.030, 0.205]	(0.009)	0.078	[-0.009, 0.165]	(0.079)
14	0.193	[0.118, 0.269]	(0.000)	0.248	[0.160, 0.336]	(0.000)
15	0.126	[0.036, 0.216]	(0.006)	0.136	[0.029, 0.243]	(0.013)
16	0.064	[-0.009, 0.137]	(0.085)	0.119	[0.034, 0.205]	(0.006)
17	0.095	[0.022, 0.168]	(0.011)	0.057	[-0.098, 0.211]	(0.471)
18	0.028	[-0.061, 0.116]	(0.539)	0.101	[-0.004, 0.205]	(0.059)
19	-0.026	[-0.107, 0.054]	(0.521)	0.044	[-0.060, 0.148]	(0.405)
20	0.002	[-0.086, 0.091]	(0.961)	-0.003	[-0.152, 0.146]	(0.968)
21	0.008	[-0.068, 0.085]	(0.831)	0.010	[-0.072, 0.092]	(0.812)
22	-0.048	[-0.135, 0.039]	(0.276)	0.017	[-0.069, 0.103]	(0.697)
23	0.003	[-0.076, 0.082]	(0.941)	0.080	[-0.073, 0.234]	(0.304)
24	-0.119	[-0.192, -0.045]	(0.002)	-0.036	[-0.120, 0.048]	(0.405)
25	0.004	[-0.088, 0.095]	(0.934)	0.034	[-0.067, 0.134]	(0.511)
26	-0.102	[-0.184, -0.020]	(0.014)	-0.012	[-0.113, 0.089]	(0.817)
27	0.066	[-0.007, 0.139]	(0.075)	0.069	[-0.050, 0.187]	(0.254)
28	0.028	[-0.051, 0.108]	(0.486)	-0.063	[-0.145, 0.019]	(0.131)
29	-0.053	[-0.123, 0.017]	(0.139)	0.029	[-0.089, 0.147]	(0.627)
30	0.040	[-0.041, 0.120]	(0.331)	-0.009	[-0.157, 0.138]	(0.902)
31	0.016	[-0.063, 0.096]	(0.684)	0.010	[-0.081, 0.102]	(0.823)
32	-0.036	[-0.118, 0.045]	(0.380)	-0.082	[-0.178, 0.014]	(0.094)
33	-0.083	[-0.160, -0.005]	(0.038)	0.094	[-0.039, 0.227]	(0.164)
34	-0.052	[-0.132, 0.027]	(0.196)	0.029	[-0.104, 0.162]	(0.669)
35	-0.050	[-0.126, 0.026]	(0.198)	-0.012	[-0.112, 0.088]	(0.819)
36	-0.075	[-0.144, -0.006]	(0.034)	-0.004	[-0.203, 0.196]	(0.972)
37	-0.069	[-0.150, 0.012]	(0.094)	-0.057	[-0.140, 0.026]	(0.180)
38	0.028	[-0.058, 0.114]	(0.518)	-0.004	[-0.118, 0.111]	(0.950)
39	0.010	[-0.070, 0.091]	(0.801)	-0.101	[-0.188, -0.015]	(0.021)
40	0.000	[-0.078, 0.078]	(0.995)	-0.083	[-0.160, -0.006]	(0.034)
41	-0.123	[-0.217, -0.029]	(0.010)	-0.140	[-0.244, -0.035]	(0.009)
42	-0.084	[-0.180, 0.011]	(0.084)	-0.116	[-0.231, -0.002]	(0.046)
43	-0.140	[-0.234, -0.045]	(0.004)	-0.143	[-0.221, -0.065]	(0.000)
44	-0.063	[-0.173, 0.047]	(0.259)	-0.003	[-0.108, 0.101]	(0.952)
45	-0.065	[-0.183, 0.053]	(0.279)	-0.084	[-0.190, 0.022]	(0.118)
46	-0.076	[-0.171, 0.020]	(0.119)	-0.026	[-0.196, 0.143]	(0.760)
47	-0.072	[-0.180, 0.036]	(0.189)	0.038	[-0.087, 0.164]	(0.550)
48	-0.029	[-0.116, 0.057]	(0.502)	0.119	[-0.124, 0.362]	(0.335)
49	-0.094	[-0.174, -0.014]	(0.021)	-0.048	[-0.151, 0.055]	(0.361)
50	0.016	[-0.052, 0.085]	(0.637)	-0.022	[-0.194, 0.151]	(0.804)
51	0.052	[-0.017, 0.122]	(0.139)	-0.103	[-0.192, -0.015]	(0.023)
52	-0.006	[-0.076, 0.064]	(0.866)	-0.001	[-0.080, 0.079]	(0.987)
N (Tweets)		292002			189562	
N (users)		320			237	

Note: The estimated coefficients on weekly indicators in 2020 with 95% confidence intervals are shown. The estimated models include user-fixed effects and week-of-year and day-of-week fixed effects.

Table S5: Estimated emotional distress level in 2020: by age group.

	18-29 yrs			30-39 yrs			40-49 yrs		
**Week**	**Coef**	**95% CI**	**p-value**	**Coef**	**95% CI**	**p-value**	**Coef**	**95% CI**	**p-value**
1	0.009	[-0.201, 0.219]	(0.934)	-0.045	[-0.171, 0.081]	(0.483)	0.024	[-0.071, 0.120]	(0.615)
2	-0.015	[-0.139, 0.108]	(0.806)	0.004	[-0.104, 0.112]	(0.941)	0.088	[-0.100, 0.277]	(0.357)
3	0.010	[-0.167, 0.188]	(0.908)	0.008	[-0.087, 0.103]	(0.869)	0.123	[-0.056, 0.301]	(0.178)
4	-0.092	[-0.254, 0.071]	(0.264)	-0.093	[-0.205, 0.020]	(0.105)	0.062	[-0.048, 0.173]	(0.269)
5	-0.037	[-0.191, 0.116]	(0.629)	0.041	[-0.056, 0.138]	(0.408)	0.241	[0.029, 0.454]	(0.026)
6	0.001	[-0.148, 0.150]	(0.986)	-0.047	[-0.138, 0.044]	(0.310)	0.072	[-0.024, 0.167]	(0.140)
7	-0.022	[-0.125, 0.080]	(0.664)	-0.008	[-0.095, 0.079]	(0.856)	0.054	[-0.119, 0.227]	(0.542)
8	0.163	[0.044, 0.283]	(0.008)	0.080	[-0.024, 0.185]	(0.132)	0.064	[-0.031, 0.158]	(0.185)
9	0.245	[0.101, 0.390]	(0.001)	0.171	[0.070, 0.272]	(0.001)	0.105	[-0.002, 0.212]	(0.054)
10	0.199	[0.064, 0.334]	(0.004)	0.050	[-0.047, 0.147]	(0.313)	0.084	[-0.050, 0.218]	(0.216)
11	-0.050	[-0.163, 0.062]	(0.377)	0.069	[-0.025, 0.163]	(0.147)	0.024	[-0.123, 0.170]	(0.752)
12	0.003	[-0.099, 0.104]	(0.959)	0.065	[-0.022, 0.153]	(0.143)	0.161	[0.028, 0.294]	(0.018)
13	0.089	[-0.054, 0.233]	(0.220)	0.104	[0.001, 0.206]	(0.047)	0.116	[0.025, 0.207]	(0.013)
14	0.091	[-0.047, 0.230]	(0.194)	0.216	[0.137, 0.295]	(0.000)	0.267	[0.174, 0.361]	(0.000)
15	0.185	[0.015, 0.356]	(0.033)	0.158	[0.073, 0.243]	(0.000)	0.071	[-0.046, 0.188]	(0.236)
16	0.112	[-0.040, 0.265]	(0.146)	0.062	[-0.014, 0.137]	(0.108)	0.101	[0.002, 0.199]	(0.045)
17	0.068	[-0.054, 0.191]	(0.271)	0.025	[-0.076, 0.126]	(0.630)	0.138	[0.005, 0.271]	(0.042)
18	0.004	[-0.138, 0.145]	(0.961)	0.036	[-0.059, 0.132]	(0.454)	0.098	[-0.015, 0.210]	(0.088)
19	0.043	[-0.070, 0.155]	(0.454)	-0.061	[-0.154, 0.033]	(0.204)	0.054	[-0.052, 0.159]	(0.316)
20	-0.037	[-0.192, 0.117]	(0.634)	-0.043	[-0.156, 0.070]	(0.459)	0.070	[-0.073, 0.213]	(0.336)
21	-0.015	[-0.117, 0.088]	(0.774)	0.046	[-0.029, 0.120]	(0.225)	-0.006	[-0.112, 0.100]	(0.912)
22	-0.014	[-0.156, 0.128]	(0.844)	-0.037	[-0.135, 0.061]	(0.456)	0.002	[-0.096, 0.100]	(0.968)
23	-0.038	[-0.164, 0.088]	(0.554)	0.037	[-0.075, 0.148]	(0.517)	0.080	[-0.057, 0.216]	(0.251)
24	-0.141	[-0.248, -0.035]	(0.010)	-0.053	[-0.146, 0.039]	(0.257)	-0.079	[-0.168, 0.010]	(0.083)
25	0.106	[-0.023, 0.234]	(0.106)	0.006	[-0.101, 0.113]	(0.911)	0.011	[-0.100, 0.121]	(0.849)
26	-0.079	[-0.192, 0.034]	(0.168)	-0.089	[-0.194, 0.017]	(0.098)	-0.024	[-0.124, 0.076]	(0.637)
27	0.104	[0.012, 0.197]	(0.028)	0.060	[-0.029, 0.148]	(0.189)	0.062	[-0.056, 0.181]	(0.300)
28	0.004	[-0.123, 0.131]	(0.947)	0.046	[-0.049, 0.140]	(0.342)	-0.066	[-0.152, 0.020]	(0.131)
29	-0.025	[-0.119, 0.068]	(0.590)	-0.110	[-0.198, -0.022]	(0.015)	0.074	[-0.038, 0.186]	(0.192)
30	0.073	[-0.066, 0.213]	(0.297)	0.000	[-0.094, 0.094]	(0.995)	0.026	[-0.115, 0.166]	(0.721)
31	0.096	[-0.021, 0.213]	(0.106)	-0.012	[-0.107, 0.084]	(0.809)	0.010	[-0.083, 0.104]	(0.832)
32	0.014	[-0.139, 0.166]	(0.859)	-0.099	[-0.198, 0.000]	(0.051)	-0.032	[-0.125, 0.061]	(0.494)
33	0.034	[-0.076, 0.144]	(0.541)	-0.066	[-0.148, 0.015]	(0.110)	0.036	[-0.100, 0.172]	(0.606)
34	-0.038	[-0.191, 0.115]	(0.622)	-0.026	[-0.109, 0.057]	(0.538)	-0.007	[-0.138, 0.124]	(0.913)
35	0.155	[0.038, 0.272]	(0.010)	-0.067	[-0.143, 0.010]	(0.088)	-0.065	[-0.169, 0.039]	(0.217)
36	0.013	[-0.118, 0.144]	(0.846)	-0.089	[-0.164, -0.015]	(0.019)	-0.026	[-0.203, 0.151]	(0.773)
37	-0.099	[-0.261, 0.063]	(0.227)	-0.032	[-0.117, 0.054]	(0.469)	-0.063	[-0.158, 0.032]	(0.191)
38	0.046	[-0.074, 0.166]	(0.448)	0.005	[-0.084, 0.095]	(0.904)	-0.007	[-0.136, 0.123]	(0.920)
39	0.029	[-0.122, 0.179]	(0.705)	0.041	[-0.050, 0.133]	(0.375)	-0.112	[-0.213, -0.011]	(0.031)
40	0.003	[-0.125, 0.131]	(0.965)	0.017	[-0.053, 0.086]	(0.636)	-0.080	[-0.183, 0.023]	(0.126)
41	0.037	[-0.117, 0.191]	(0.631)	-0.133	[-0.233, -0.032]	(0.010)	-0.184	[-0.299, -0.069]	(0.002)
42	-0.067	[-0.194, 0.060]	(0.297)	-0.058	[-0.155, 0.038]	(0.232)	-0.144	[-0.275, -0.013]	(0.031)
43	-0.082	[-0.212, 0.048]	(0.212)	-0.100	[-0.198, -0.003]	(0.044)	-0.198	[-0.301, -0.095]	(0.000)
44	0.013	[-0.224, 0.249]	(0.916)	-0.023	[-0.108, 0.062]	(0.600)	-0.083	[-0.218, 0.052]	(0.229)
45	-0.108	[-0.248, 0.033]	(0.130)	-0.010	[-0.120, 0.100]	(0.860)	-0.130	[-0.281, 0.021]	(0.091)
46	0.058	[-0.052, 0.168]	(0.299)	-0.105	[-0.193, -0.017]	(0.019)	-0.062	[-0.248, 0.123]	(0.507)
47	0.010	[-0.118, 0.138]	(0.877)	-0.007	[-0.103, 0.089]	(0.885)	-0.050	[-0.213, 0.113]	(0.548)
48	0.031	[-0.113, 0.175]	(0.671)	0.036	[-0.075, 0.146]	(0.526)	0.029	[-0.196, 0.255]	(0.797)
49	-0.038	[-0.190, 0.113]	(0.616)	-0.092	[-0.182, -0.002]	(0.046)	-0.073	[-0.177, 0.032]	(0.173)
50	0.044	[-0.059, 0.148]	(0.397)	0.000	[-0.067, 0.067]	(0.994)	-0.014	[-0.167, 0.139]	(0.857)
51	0.055	[-0.043, 0.153]	(0.265)	-0.009	[-0.102, 0.085]	(0.856)	-0.033	[-0.120, 0.053]	(0.449)
52	0.064	[-0.058, 0.187]	(0.300)	0.009	[-0.069, 0.088]	(0.813)	-0.043	[-0.128, 0.042]	(0.321)
N (Tweets)		81883			200424			205977	
N (users)		83			228			249	

Note: The estimated coefficients on weekly indicators in 2020 with 95% confidence intervals are shown. The estimated models include user-fixed effects and week-of-year and day-of-week fixed effects.

Table S6: Estimated emotional distress level in 2020: by income.

	Income: below 6 million yen			Income: 6 milion yen or higher		
Week	Coef	95% CI	p-value	Coef	95% CI	p-value
1	-0.001	[-0.103, 0.101]	(0.985)	0.002	[-0.119, 0.124]	(0.972)
2	0.085	[-0.047, 0.216]	(0.205)	-0.058	[-0.152, 0.036]	(0.225)
3	0.074	[-0.053, 0.201]	(0.254)	0.006	[-0.082, 0.093]	(0.897)
4	-0.039	[-0.124, 0.045]	(0.361)	-0.021	[-0.166, 0.124]	(0.772)
5	0.109	[-0.031, 0.249]	(0.128)	0.084	[-0.026, 0.194]	(0.133)
6	0.066	[-0.009, 0.141]	(0.084)	-0.097	[-0.191, -0.002]	(0.046)
7	0.075	[-0.027, 0.178]	(0.151)	-0.105	[-0.219, 0.009]	(0.072)
8	0.114	[0.045, 0.184]	(0.001)	0.039	[-0.083, 0.161]	(0.529)
9	0.181	[0.103, 0.259]	(0.000)	0.114	[-0.005, 0.232]	(0.060)
10	0.132	[0.038, 0.226]	(0.006)	0.010	[-0.099, 0.119]	(0.860)
11	0.053	[-0.048, 0.154]	(0.300)	-0.016	[-0.127, 0.095]	(0.778)
12	0.128	[0.028, 0.229]	(0.012)	0.042	[-0.041, 0.124]	(0.321)
13	0.140	[0.067, 0.214]	(0.000)	0.040	[-0.068, 0.149]	(0.465)
14	0.256	[0.189, 0.324]	(0.000)	0.151	[0.046, 0.255]	(0.005)
15	0.129	[0.048, 0.210]	(0.002)	0.126	[0.003, 0.248]	(0.045)
16	0.096	[0.027, 0.165]	(0.006)	0.061	[-0.034, 0.156]	(0.208)
17	0.097	[0.004, 0.191]	(0.040)	0.052	[-0.066, 0.170]	(0.385)
18	0.099	[0.024, 0.173]	(0.009)	-0.010	[-0.124, 0.105]	(0.867)
19	0.003	[-0.073, 0.078]	(0.947)	0.002	[-0.115, 0.119]	(0.968)
20	-0.005	[-0.103, 0.094]	(0.922)	0.013	[-0.116, 0.141]	(0.848)
21	0.030	[-0.040, 0.101]	(0.399)	-0.023	[-0.112, 0.066]	(0.612)
22	-0.022	[-0.103, 0.059]	(0.596)	-0.016	[-0.109, 0.078]	(0.745)
23	0.102	[0.012, 0.191]	(0.026)	-0.083	[-0.196, 0.029]	(0.147)
24	-0.091	[-0.158, -0.023]	(0.008)	-0.056	[-0.161, 0.049]	(0.298)
25	0.035	[-0.053, 0.123]	(0.437)	0.009	[-0.094, 0.113]	(0.858)
26	-0.059	[-0.135, 0.018]	(0.131)	-0.068	[-0.181, 0.045]	(0.238)
27	0.082	[-0.002, 0.166]	(0.057)	0.043	[-0.049, 0.135]	(0.362)
28	0.017	[-0.049, 0.083]	(0.615)	-0.059	[-0.169, 0.051]	(0.294)
29	-0.038	[-0.118, 0.043]	(0.359)	0.028	[-0.073, 0.129]	(0.583)
30	0.036	[-0.065, 0.137]	(0.482)	-0.000	[-0.102, 0.101]	(0.993)
31	0.028	[-0.049, 0.104]	(0.475)	-0.003	[-0.101, 0.095]	(0.957)
32	-0.016	[-0.092, 0.061]	(0.688)	-0.128	[-0.232, -0.025]	(0.016)
33	0.008	[-0.089, 0.105]	(0.870)	-0.044	[-0.146, 0.058]	(0.395)
34	-0.011	[-0.101, 0.079]	(0.808)	-0.042	[-0.146, 0.062]	(0.424)
35	-0.052	[-0.127, 0.022]	(0.165)	-0.003	[-0.101, 0.094]	(0.947)
36	-0.018	[-0.156, 0.120]	(0.800)	-0.089	[-0.169, -0.010]	(0.027)
37	-0.049	[-0.128, 0.030]	(0.227)	-0.067	[-0.158, 0.024]	(0.146)
38	0.034	[-0.058, 0.126]	(0.466)	-0.039	[-0.143, 0.066]	(0.466)
39	-0.002	[-0.079, 0.076]	(0.963)	-0.065	[-0.172, 0.042]	(0.235)
40	0.017	[-0.044, 0.078]	(0.589)	-0.107	[-0.213, -0.001]	(0.048)
41	-0.140	[-0.227, -0.053]	(0.002)	-0.100	[-0.219, 0.019]	(0.098)
42	-0.025	[-0.102, 0.051]	(0.516)	-0.231	[-0.363, -0.098]	(0.001)
43	-0.127	[-0.208, -0.047]	(0.002)	-0.166	[-0.267, -0.064]	(0.001)
44	0.016	[-0.063, 0.096]	(0.685)	-0.125	[-0.275, 0.024]	(0.100)
45	-0.063	[-0.147, 0.022]	(0.145)	-0.088	[-0.265, 0.090]	(0.331)
46	-0.055	[-0.168, 0.059]	(0.344)	-0.073	[-0.206, 0.060]	(0.282)
47	0.022	[-0.070, 0.113]	(0.640)	-0.098	[-0.252, 0.057]	(0.215)
48	0.062	[-0.088, 0.213]	(0.416)	-0.032	[-0.170, 0.105]	(0.642)
49	-0.057	[-0.135, 0.020]	(0.148)	-0.105	[-0.213, 0.002]	(0.055)
50	0.034	[-0.075, 0.143]	(0.538)	-0.059	[-0.153, 0.036]	(0.221)
51	-0.041	[-0.111, 0.029]	(0.248)	0.054	[-0.034, 0.142]	(0.226)
52	-0.006	[-0.072, 0.061]	(0.866)	0.000	[-0.087, 0.087]	(1.000)
N (Tweets)		317767			170517	
N (users)		350			210	

Note: The estimated coefficients on weekly indicators in 2020 with 95% confidence intervals are shown. The estimated models include user-fixed effects and week-of-year and day-of-week fixed effects.

Table S7: Estimated emotional distress level in 2020: by the type of employment and employment status.

	Part-time/temporary worker or unemployed			Permanent employee			Not in the labor force		
Week	Coef	95% CI	p-value	Coef	95% CI	p-value	Coef	95% CI	p-value
1	-0.034	[-0.166, 0.099]	(0.617)	0.062	[-0.031, 0.155]	(0.191)	-0.102	[-0.357, 0.153]	(0.428)
2	0.117	[-0.098, 0.331]	(0.285)	0.023	[-0.053, 0.099]	(0.550)	-0.066	[-0.262, 0.131]	(0.508)
3	0.116	[-0.078, 0.310]	(0.241)	-0.003	[-0.077, 0.072]	(0.942)	0.076	[-0.089, 0.240]	(0.362)
4	-0.063	[-0.207, 0.081]	(0.388)	-0.004	[-0.093, 0.084]	(0.923)	-0.055	[-0.266, 0.156]	(0.606)
5	0.208	[-0.016, 0.431]	(0.068)	0.079	[-0.006, 0.164]	(0.068)	-0.099	[-0.229, 0.031]	(0.133)
6	0.096	[-0.020, 0.213]	(0.105)	-0.022	[-0.096, 0.051]	(0.546)	-0.101	[-0.268, 0.066]	(0.234)
7	0.133	[-0.042, 0.308]	(0.135)	-0.060	[-0.150, 0.029]	(0.186)	-0.056	[-0.183, 0.071]	(0.383)
8	0.145	[0.041, 0.248]	(0.007)	0.051	[-0.039, 0.141]	(0.269)	0.122	[-0.045, 0.289]	(0.150)
9	0.154	[0.056, 0.251]	(0.002)	0.180	[0.080, 0.281]	(0.000)	0.027	[-0.100, 0.154]	(0.673)
10	0.121	[-0.040, 0.283]	(0.141)	0.100	[0.015, 0.185]	(0.022)	0.008	[-0.120, 0.136]	(0.905)
11	0.138	[-0.018, 0.295]	(0.082)	-0.010	[-0.099, 0.080]	(0.832)	-0.071	[-0.224, 0.083]	(0.363)
12	0.184	[0.012, 0.356]	(0.036)	0.079	[0.016, 0.142]	(0.014)	-0.047	[-0.188, 0.093]	(0.506)
13	0.161	[0.054, 0.269]	(0.003)	0.067	[-0.024, 0.158]	(0.148)	0.084	[-0.058, 0.225]	(0.243)
14	0.304	[0.206, 0.401]	(0.000)	0.142	[0.069, 0.216]	(0.000)	0.286	[0.144, 0.429]	(0.000)
15	0.230	[0.115, 0.345]	(0.000)	0.096	[-0.003, 0.195]	(0.059)	0.032	[-0.123, 0.188]	(0.679)
16	0.134	[0.022, 0.247]	(0.019)	0.066	[-0.006, 0.138]	(0.073)	0.034	[-0.078, 0.147]	(0.546)
17	0.150	[0.007, 0.294]	(0.040)	0.088	[-0.004, 0.179]	(0.061)	-0.064	[-0.238, 0.111]	(0.471)
18	0.189	[0.082, 0.296]	(0.001)	0.008	[-0.082, 0.098]	(0.864)	-0.077	[-0.225, 0.071]	(0.303)
19	0.116	[0.026, 0.206]	(0.012)	-0.021	[-0.098, 0.056]	(0.593)	-0.160	[-0.350, 0.030]	(0.097)
20	0.084	[-0.078, 0.246]	(0.308)	-0.035	[-0.130, 0.059]	(0.460)	-0.088	[-0.239, 0.064]	(0.252)
21	0.002	[-0.100, 0.103]	(0.973)	0.072	[0.001, 0.143]	(0.047)	-0.075	[-0.242, 0.093]	(0.377)
22	-0.011	[-0.142, 0.120]	(0.870)	0.002	[-0.069, 0.074]	(0.947)	-0.076	[-0.240, 0.087]	(0.356)
23	0.093	[-0.070, 0.256]	(0.261)	0.002	[-0.083, 0.087]	(0.963)	0.078	[-0.093, 0.249]	(0.367)
24	-0.120	[-0.217, -0.022]	(0.016)	-0.051	[-0.131, 0.029]	(0.214)	-0.062	[-0.213, 0.090]	(0.421)
25	0.045	[-0.093, 0.184]	(0.517)	0.018	[-0.064, 0.100]	(0.659)	-0.030	[-0.210, 0.150]	(0.743)
26	-0.119	[-0.230, -0.007]	(0.037)	-0.010	[-0.095, 0.075]	(0.822)	-0.129	[-0.245, -0.013]	(0.030)
27	0.109	[-0.019, 0.238]	(0.095)	0.041	[-0.038, 0.120]	(0.307)	0.038	[-0.095, 0.171]	(0.569)
28	-0.018	[-0.125, 0.089]	(0.743)	-0.018	[-0.095, 0.059]	(0.645)	-0.018	[-0.148, 0.112]	(0.783)
29	0.022	[-0.104, 0.149]	(0.728)	-0.021	[-0.101, 0.059]	(0.608)	-0.065	[-0.180, 0.051]	(0.268)
30	0.002	[-0.181, 0.185]	(0.983)	0.035	[-0.044, 0.114]	(0.379)	-0.001	[-0.101, 0.100]	(0.988)
31	0.122	[0.010, 0.235]	(0.034)	-0.011	[-0.088, 0.066]	(0.781)	-0.152	[-0.299, -0.005]	(0.043)
32	-0.040	[-0.156, 0.076]	(0.500)	-0.084	[-0.171, 0.004]	(0.061)	-0.021	[-0.142, 0.099]	(0.727)
33	0.013	[-0.134, 0.161]	(0.858)	0.005	[-0.067, 0.077]	(0.894)	-0.210	[-0.359, -0.062]	(0.006)
34	0.009	[-0.142, 0.160]	(0.910)	-0.027	[-0.101, 0.046]	(0.465)	-0.070	[-0.217, 0.076]	(0.343)
35	-0.017	[-0.140, 0.107]	(0.791)	-0.022	[-0.085, 0.041]	(0.491)	-0.113	[-0.264, 0.038]	(0.140)
36	0.026	[-0.207, 0.259]	(0.824)	-0.123	[-0.189, -0.057]	(0.000)	0.052	[-0.059, 0.162]	(0.356)
37	-0.060	[-0.179, 0.059]	(0.324)	-0.054	[-0.131, 0.022]	(0.162)	-0.077	[-0.218, 0.064]	(0.280)
38	0.024	[-0.118, 0.166]	(0.739)	0.036	[-0.040, 0.111]	(0.352)	-0.158	[-0.350, 0.034]	(0.106)
39	-0.009	[-0.111, 0.094]	(0.868)	-0.018	[-0.112, 0.077]	(0.711)	-0.087	[-0.273, 0.098]	(0.350)
40	-0.001	[-0.086, 0.085]	(0.988)	-0.018	[-0.089, 0.053]	(0.615)	-0.124	[-0.315, 0.067]	(0.201)
41	-0.094	[-0.209, 0.022]	(0.110)	-0.129	[-0.227, -0.031]	(0.010)	-0.224	[-0.461, 0.014]	(0.065)
42	-0.046	[-0.188, 0.097]	(0.528)	-0.096	[-0.163, -0.030]	(0.005)	-0.268	[-0.544, 0.007]	(0.056)
43	-0.204	[-0.304, -0.105]	(0.000)	-0.063	[-0.135, 0.010]	(0.090)	-0.290	[-0.512, -0.068]	(0.011)
44	0.061	[-0.057, 0.178]	(0.312)	-0.077	[-0.179, 0.025]	(0.139)	-0.129	[-0.435, 0.176]	(0.402)
45	-0.042	[-0.166, 0.083]	(0.511)	-0.052	[-0.154, 0.051]	(0.323)	-0.217	[-0.510, 0.075]	(0.142)
46	-0.005	[-0.185, 0.175]	(0.957)	-0.065	[-0.144, 0.014]	(0.105)	-0.218	[-0.477, 0.041]	(0.098)
47	0.016	[-0.137, 0.170]	(0.834)	-0.003	[-0.076, 0.070]	(0.932)	-0.124	[-0.453, 0.204]	(0.454)
48	0.105	[-0.138, 0.347]	(0.396)	-0.007	[-0.103, 0.090]	(0.889)	-0.073	[-0.319, 0.172]	(0.553)
49	-0.054	[-0.169, 0.062]	(0.359)	-0.068	[-0.149, 0.013]	(0.097)	-0.117	[-0.311, 0.076]	(0.232)
50	0.098	[-0.092, 0.288]	(0.311)	-0.056	[-0.120, 0.008]	(0.089)	-0.031	[-0.202, 0.141]	(0.724)
51	-0.045	[-0.159, 0.069]	(0.439)	0.008	[-0.060, 0.075]	(0.823)	0.052	[-0.099, 0.202]	(0.495)
52	0.013	[-0.074, 0.099]	(0.773)	0.024	[-0.050, 0.098]	(0.523)	-0.124	[-0.268, 0.020]	(0.090)
N (Tweets)		170269			238218			68215	
N (users)		174			294			79	

Note: The estimated coefficients on weekly indicators in 2020 with 95% confidence intervals are shown. The estimated models include user-fixed effects and week-of-year and day-of-week fixed effects.

Table S8: Estimated emotional distress level in 2020: by the presence of depressive symptoms.

	No depressive symptoms			With depressive symptoms		
**Week**	**Coef**	**95% CI**	**p-value**	**Coef**	**95% CI**	**p-value**
1	-0.023	[-0.123, 0.077]	(0.657)	0.050	[-0.065, 0.166]	(0.393)
2	0.019	[-0.061, 0.098]	(0.645)	0.071	[-0.156, 0.297]	(0.539)
3	0.004	[-0.073, 0.082]	(0.914)	0.152	[-0.064, 0.368]	(0.166)
4	-0.036	[-0.123, 0.051]	(0.415)	-0.025	[-0.165, 0.114]	(0.719)
5	0.080	[0.009, 0.151]	(0.027)	0.130	[-0.098, 0.357]	(0.261)
6	0.024	[-0.050, 0.098]	(0.530)	-0.015	[-0.121, 0.092]	(0.787)
7	-0.038	[-0.116, 0.040]	(0.339)	0.123	[-0.074, 0.320]	(0.220)
8	0.086	[0.011, 0.160]	(0.024)	0.099	[-0.015, 0.213]	(0.089)
9	0.175	[0.097, 0.252]	(0.000)	0.130	[0.016, 0.243]	(0.026)
10	0.071	[-0.008, 0.150]	(0.076)	0.128	[-0.028, 0.284]	(0.108)
11	0.075	[-0.023, 0.173]	(0.133)	-0.065	[-0.174, 0.045]	(0.246)
12	0.088	[0.021, 0.156]	(0.010)	0.111	[-0.055, 0.276]	(0.188)
13	0.127	[0.045, 0.208]	(0.003)	0.062	[-0.023, 0.147]	(0.152)
14	0.186	[0.119, 0.254]	(0.000)	0.300	[0.198, 0.401]	(0.000)
15	0.115	[0.036, 0.194]	(0.005)	0.162	[0.029, 0.295]	(0.018)
16	0.092	[0.030, 0.155]	(0.004)	0.070	[-0.045, 0.185]	(0.232)
17	0.050	[-0.035, 0.135]	(0.251)	0.147	[0.004, 0.289]	(0.044)
18	0.022	[-0.056, 0.101]	(0.580)	0.135	[0.019, 0.251]	(0.023)
19	0.011	[-0.068, 0.091]	(0.778)	-0.013	[-0.114, 0.088]	(0.805)
20	-0.026	[-0.111, 0.060]	(0.552)	0.056	[-0.110, 0.223]	(0.507)
21	0.039	[-0.026, 0.105]	(0.241)	-0.038	[-0.142, 0.066]	(0.472)
22	-0.016	[-0.090, 0.058]	(0.666)	-0.024	[-0.141, 0.092]	(0.679)
23	0.015	[-0.061, 0.092]	(0.695)	0.089	[-0.072, 0.249]	(0.276)
24	-0.116	[-0.183, -0.049]	(0.001)	-0.013	[-0.110, 0.084]	(0.790)
25	0.025	[-0.043, 0.093]	(0.465)	0.026	[-0.140, 0.192]	(0.756)
26	-0.050	[-0.127, 0.027]	(0.199)	-0.079	[-0.182, 0.024]	(0.133)
27	0.052	[-0.016, 0.121]	(0.133)	0.105	[-0.033, 0.243]	(0.134)
28	-0.003	[-0.075, 0.069]	(0.937)	-0.014	[-0.108, 0.080]	(0.771)
29	-0.051	[-0.121, 0.020]	(0.160)	0.054	[-0.071, 0.180]	(0.396)
30	-0.001	[-0.076, 0.074]	(0.977)	0.090	[-0.094, 0.274]	(0.337)
31	0.017	[-0.055, 0.089]	(0.650)	0.018	[-0.094, 0.130]	(0.752)
32	-0.056	[-0.128, 0.017]	(0.130)	-0.055	[-0.171, 0.060]	(0.344)
33	0.005	[-0.066, 0.077]	(0.887)	-0.037	[-0.194, 0.119]	(0.637)
34	-0.047	[-0.116, 0.023]	(0.191)	0.035	[-0.122, 0.191]	(0.663)
35	-0.044	[-0.113, 0.025]	(0.208)	-0.009	[-0.118, 0.099]	(0.864)
36	-0.098	[-0.159, -0.037]	(0.002)	0.063	[-0.178, 0.303]	(0.608)
37	-0.065	[-0.131, 0.001]	(0.054)	-0.038	[-0.163, 0.087]	(0.552)
38	-0.013	[-0.087, 0.062]	(0.739)	0.048	[-0.100, 0.196]	(0.520)
39	-0.055	[-0.131, 0.020]	(0.149)	0.036	[-0.079, 0.150]	(0.537)
40	-0.049	[-0.119, 0.021]	(0.172)	0.012	[-0.077, 0.101]	(0.792)
41	-0.125	[-0.213, -0.037]	(0.005)	-0.125	[-0.244, -0.007]	(0.038)
42	-0.154	[-0.237, -0.072]	(0.000)	0.024	[-0.112, 0.160]	(0.731)
43	-0.125	[-0.200, -0.050]	(0.001)	-0.173	[-0.294, -0.052]	(0.005)
44	-0.034	[-0.131, 0.063]	(0.494)	-0.045	[-0.174, 0.085]	(0.498)
45	-0.109	[-0.202, -0.016]	(0.021)	0.011	[-0.151, 0.173]	(0.893)
46	-0.071	[-0.155, 0.013]	(0.098)	-0.036	[-0.238, 0.166]	(0.725)
47	-0.047	[-0.137, 0.043]	(0.310)	0.042	[-0.129, 0.213]	(0.627)
48	-0.030	[-0.112, 0.052]	(0.471)	0.145	[-0.124, 0.413]	(0.290)
49	-0.097	[-0.172, -0.022]	(0.012)	-0.021	[-0.139, 0.098]	(0.734)
50	-0.021	[-0.085, 0.043]	(0.522)	0.043	[-0.153, 0.239]	(0.665)
51	0.004	[-0.062, 0.070]	(0.911)	-0.041	[-0.144, 0.062]	(0.434)
52	-0.002	[-0.066, 0.062]	(0.952)	-0.007	[-0.100, 0.086]	(0.877)
N (Tweets)		331227			157057	
N (users)		384			176	

Note: The estimated coefficients on weekly indicators in 2020 with 95% confidence intervals are shown. The estimated models include user-fixed effects and week-of-year and day-of-week fixed effects.

Table S9: Estimated emotional distress level in 2020: by suicidal ideation.

	No suicidal ideation			With suicidal ideation		
**Week**	**Coef**	**95% CI**	**p-value**	**Coef**	**95% CI**	**p-value**
1	0.021	[-0.069, 0.112]	(0.643)	-0.030	[-0.170, 0.110]	(0.674)
2	0.051	[-0.031, 0.133]	(0.221)	0.011	[-0.192, 0.214]	(0.916)
3	0.037	[-0.039, 0.112]	(0.339)	0.070	[-0.112, 0.252]	(0.448)
4	-0.011	[-0.094, 0.073]	(0.800)	-0.065	[-0.199, 0.069]	(0.338)
5	0.064	[-0.008, 0.137]	(0.082)	0.145	[-0.061, 0.351]	(0.167)
6	-0.021	[-0.098, 0.056]	(0.590)	0.059	[-0.040, 0.157]	(0.242)
7	-0.048	[-0.127, 0.030]	(0.225)	0.111	[-0.054, 0.275]	(0.185)
8	0.064	[-0.016, 0.144]	(0.114)	0.128	[0.031, 0.225]	(0.010)
9	0.132	[0.054, 0.210]	(0.001)	0.206	[0.091, 0.320]	(0.001)
10	0.068	[-0.013, 0.148]	(0.098)	0.122	[-0.020, 0.263]	(0.091)
11	0.094	[-0.008, 0.197]	(0.071)	-0.084	[-0.182, 0.014]	(0.092)
12	0.075	[0.004, 0.145]	(0.037)	0.126	[-0.013, 0.265]	(0.075)
13	0.109	[0.023, 0.196]	(0.014)	0.095	[0.012, 0.178]	(0.026)
14	0.184	[0.113, 0.255]	(0.000)	0.286	[0.194, 0.378]	(0.000)
15	0.102	[0.021, 0.184]	(0.014)	0.183	[0.061, 0.305]	(0.003)
16	0.085	[0.022, 0.149]	(0.009)	0.083	[-0.022, 0.187]	(0.119)
17	0.037	[-0.048, 0.123]	(0.388)	0.158	[0.025, 0.291]	(0.021)
18	0.006	[-0.075, 0.086]	(0.892)	0.145	[0.039, 0.251]	(0.008)
19	-0.010	[-0.090, 0.070]	(0.808)	0.021	[-0.078, 0.119]	(0.676)
20	-0.042	[-0.132, 0.047]	(0.356)	0.068	[-0.079, 0.216]	(0.363)
21	0.019	[-0.043, 0.080]	(0.552)	0.004	[-0.101, 0.109]	(0.944)
22	-0.048	[-0.125, 0.029]	(0.223)	0.030	[-0.075, 0.135]	(0.575)
23	-0.015	[-0.092, 0.062]	(0.707)	0.126	[-0.014, 0.266]	(0.078)
24	-0.133	[-0.206, -0.060]	(0.000)	-0.005	[-0.091, 0.080]	(0.902)
25	-0.006	[-0.081, 0.069]	(0.869)	0.076	[-0.052, 0.203]	(0.244)
26	-0.062	[-0.146, 0.021]	(0.142)	-0.067	[-0.166, 0.032]	(0.182)
27	0.034	[-0.037, 0.104]	(0.346)	0.124	[0.001, 0.247]	(0.048)
28	0.014	[-0.060, 0.088]	(0.717)	-0.040	[-0.130, 0.051]	(0.387)
29	-0.080	[-0.150, -0.009]	(0.027)	0.087	[-0.027, 0.201]	(0.133)
30	-0.024	[-0.100, 0.052]	(0.540)	0.124	[-0.039, 0.287]	(0.134)
31	0.018	[-0.056, 0.092]	(0.633)	0.014	[-0.090, 0.118]	(0.791)
32	-0.088	[-0.161, -0.015]	(0.018)	-0.004	[-0.114, 0.106]	(0.945)
33	-0.025	[-0.098, 0.048]	(0.501)	0.019	[-0.124, 0.162]	(0.796)
34	-0.052	[-0.123, 0.019]	(0.151)	0.036	[-0.107, 0.178]	(0.622)
35	-0.045	[-0.116, 0.027]	(0.217)	-0.011	[-0.111, 0.090]	(0.836)
36	-0.100	[-0.165, -0.035]	(0.003)	0.054	[-0.173, 0.281]	(0.642)
37	-0.099	[-0.167, -0.031]	(0.005)	0.021	[-0.092, 0.134]	(0.712)
38	-0.035	[-0.114, 0.043]	(0.376)	0.088	[-0.048, 0.224]	(0.204)
39	-0.074	[-0.153, 0.004]	(0.063)	0.055	[-0.050, 0.160]	(0.301)
40	-0.047	[-0.122, 0.028]	(0.221)	0.002	[-0.077, 0.081]	(0.963)
41	-0.125	[-0.215, -0.035]	(0.006)	-0.135	[-0.242, -0.028]	(0.014)
42	-0.146	[-0.230, -0.062]	(0.001)	-0.015	[-0.143, 0.113]	(0.815)
43	-0.133	[-0.212, -0.054]	(0.001)	-0.153	[-0.260, -0.045]	(0.006)
44	-0.066	[-0.166, 0.035]	(0.200)	0.008	[-0.106, 0.122]	(0.889)
45	-0.141	[-0.238, -0.044]	(0.005)	0.052	[-0.093, 0.197]	(0.482)
46	-0.100	[-0.190, -0.011]	(0.028)	0.014	[-0.168, 0.196]	(0.877)
47	-0.036	[-0.133, 0.061]	(0.469)	0.012	[-0.139, 0.164]	(0.872)
48	-0.035	[-0.118, 0.048]	(0.411)	0.131	[-0.114, 0.375]	(0.292)
49	-0.105	[-0.182, -0.029]	(0.007)	-0.017	[-0.130, 0.095]	(0.760)
50	-0.037	[-0.099, 0.026]	(0.247)	0.062	[-0.121, 0.245]	(0.503)
51	-0.007	[-0.077, 0.063]	(0.849)	-0.015	[-0.108, 0.079]	(0.760)
52	-0.025	[-0.092, 0.041]	(0.454)	0.034	[-0.051, 0.120]	(0.432)
N (Tweets)		307018			181266	
N (users)		391			169	

Note: The estimated coefficients on weekly indicators in 2020 with 95% confidence intervals are shown. The estimated models include user-fixed effects and week-of-year and day-of-week fixed effects.

Table S10: Estimated emotional distress level in 2020: by loneliness.

	Not lonely			Moderate to high loneliness		
**Week**	**Coef**	**95% CI**	**p-value**	**Coef**	**95% CI**	**p-value**
1	-0.019	[-0.126, 0.088]	(0.725)	0.018	[-0.093, 0.129]	(0.747)
2	0.034	[-0.079, 0.147]	(0.549)	0.035	[-0.110, 0.180]	(0.637)
3	0.002	[-0.082, 0.085]	(0.972)	0.098	[-0.051, 0.248]	(0.197)
4	-0.016	[-0.103, 0.071]	(0.716)	-0.046	[-0.166, 0.074]	(0.451)
5	0.067	[-0.015, 0.149]	(0.111)	0.130	[-0.041, 0.301]	(0.136)
6	-0.011	[-0.104, 0.083]	(0.824)	0.030	[-0.050, 0.109]	(0.460)
7	-0.031	[-0.128, 0.067]	(0.539)	0.051	[-0.078, 0.180]	(0.436)
8	0.072	[-0.018, 0.161]	(0.116)	0.109	[0.024, 0.194]	(0.012)
9	0.180	[0.082, 0.278]	(0.000)	0.143	[0.056, 0.230]	(0.001)
10	0.077	[-0.015, 0.170]	(0.100)	0.100	[-0.012, 0.211]	(0.080)
11	0.108	[-0.014, 0.230]	(0.082)	-0.048	[-0.134, 0.037]	(0.268)
12	0.102	[0.014, 0.190]	(0.023)	0.081	[-0.017, 0.179]	(0.106)
13	0.114	[0.022, 0.205]	(0.015)	0.095	[0.011, 0.179]	(0.026)
14	0.187	[0.111, 0.264]	(0.000)	0.249	[0.163, 0.336]	(0.000)
15	0.075	[-0.013, 0.163]	(0.095)	0.180	[0.079, 0.281]	(0.001)
16	0.064	[-0.011, 0.140]	(0.094)	0.105	[0.018, 0.191]	(0.018)
17	0.097	[0.000, 0.194]	(0.049)	0.064	[-0.046, 0.174]	(0.255)
18	0.001	[-0.106, 0.107]	(0.991)	0.107	[0.028, 0.185]	(0.008)
19	0.013	[-0.072, 0.098]	(0.767)	-0.007	[-0.099, 0.085]	(0.887)
20	-0.006	[-0.111, 0.099]	(0.913)	0.007	[-0.112, 0.125]	(0.913)
21	0.039	[-0.049, 0.126]	(0.385)	-0.009	[-0.079, 0.062]	(0.809)
22	-0.016	[-0.110, 0.078]	(0.735)	-0.018	[-0.101, 0.066]	(0.676)
23	0.029	[-0.061, 0.120]	(0.526)	0.051	[-0.068, 0.171]	(0.399)
24	-0.127	[-0.213, -0.042]	(0.004)	-0.038	[-0.111, 0.034]	(0.299)
25	-0.010	[-0.097, 0.076]	(0.816)	0.063	[-0.042, 0.168]	(0.240)
26	-0.047	[-0.141, 0.047]	(0.322)	-0.069	[-0.150, 0.013]	(0.098)
27	0.043	[-0.039, 0.126]	(0.304)	0.096	[-0.001, 0.194]	(0.053)
28	0.014	[-0.073, 0.101]	(0.749)	-0.025	[-0.100, 0.050]	(0.511)
29	-0.026	[-0.106, 0.054]	(0.525)	-0.008	[-0.106, 0.090]	(0.878)
30	-0.015	[-0.106, 0.077]	(0.755)	0.062	[-0.060, 0.184]	(0.317)
31	0.027	[-0.061, 0.115]	(0.545)	0.009	[-0.074, 0.093]	(0.824)
32	-0.033	[-0.120, 0.053]	(0.451)	-0.082	[-0.170, 0.005]	(0.066)
33	0.023	[-0.060, 0.105]	(0.585)	-0.035	[-0.152, 0.081]	(0.550)
34	-0.034	[-0.121, 0.053]	(0.443)	-0.011	[-0.117, 0.095]	(0.837)
35	-0.031	[-0.109, 0.046]	(0.427)	-0.033	[-0.120, 0.054]	(0.456)
36	-0.123	[-0.200, -0.045]	(0.002)	0.008	[-0.138, 0.155]	(0.910)
37	-0.089	[-0.164, -0.014]	(0.021)	-0.030	[-0.121, 0.061]	(0.516)
38	0.013	[-0.069, 0.095]	(0.756)	0.004	[-0.106, 0.114]	(0.943)
39	-0.029	[-0.125, 0.068]	(0.559)	-0.024	[-0.111, 0.064]	(0.597)
40	-0.014	[-0.080, 0.052]	(0.676)	-0.039	[-0.126, 0.047]	(0.373)
41	-0.084	[-0.180, 0.013]	(0.090)	-0.160	[-0.259, -0.062]	(0.001)
42	-0.121	[-0.195, -0.047]	(0.001)	-0.080	[-0.202, 0.042]	(0.198)
43	-0.102	[-0.175, -0.028]	(0.007)	-0.177	[-0.277, -0.077]	(0.001)
44	0.036	[-0.040, 0.112]	(0.351)	-0.104	[-0.231, 0.023]	(0.108)
45	-0.060	[-0.146, 0.026]	(0.169)	-0.080	[-0.215, 0.056]	(0.248)
46	-0.039	[-0.113, 0.035]	(0.296)	-0.079	[-0.232, 0.074]	(0.313)
47	0.019	[-0.059, 0.098]	(0.626)	-0.056	[-0.194, 0.083]	(0.430)
48	0.039	[-0.045, 0.123]	(0.367)	0.020	[-0.174, 0.214]	(0.837)
49	-0.052	[-0.138, 0.035]	(0.244)	-0.093	[-0.185, -0.002]	(0.045)
50	-0.028	[-0.099, 0.042]	(0.434)	0.028	[-0.107, 0.162]	(0.687)
51	0.028	[-0.050, 0.106]	(0.484)	-0.044	[-0.125, 0.036]	(0.278)
52	-0.004	[-0.088, 0.080]	(0.923)	-0.003	[-0.069, 0.063]	(0.923)
N (Tweets)		255327			232957	
N (users)		276			284	

Note: The estimated coefficients on weekly indicators in 2020 with 95% confidence intervals are shown. The estimated models include user-fixed effects and week-of-year and day-of-week fixed effects.
